# pVACview: an interactive visualization tool for efficient neoantigen prioritization and selection

**DOI:** 10.1186/s13073-024-01384-7

**Published:** 2024-11-14

**Authors:** Huiming Xia, My H. Hoang, Evelyn Schmidt, Susanna Kiwala, Joshua McMichael, Zachary L. Skidmore, Bryan Fisk, Jonathan J. Song, Jasreet Hundal, Thomas Mooney, Jason R. Walker, S. Peter Goedegebuure, Christopher A. Miller, William E. Gillanders, Obi L. Griffith, Malachi Griffith

**Affiliations:** 1 Division of Oncology, Department of Medicine, Washington University School of Medicine, St. Louis, MO USA; 2 McDonnell Genome Institute, Washington University School of Medicine, St. Louis, MO USA; 3 Department of Surgery, Washington University School of Medicine, St. Louis, MO USA; 4 Siteman Cancer Center, Washington University School of Medicine, St. Louis, MO USA; 5 Department of Genetics, Washington University School of Medicine, St. Louis, MO USA

**Keywords:** Neoantigen, Data visualization, Vaccine design, Pipeline, Prioritization, Cancer immunotherapy

## Abstract

**Background:**

Neoantigen-targeting therapies including personalized vaccines have shown promise in the treatment of cancers, particularly when used in combination with checkpoint blockade therapy. At least 100 clinical trials involving these therapies have been initiated globally. Accurate identification and prioritization of neoantigens is crucial for designing these trials, predicting treatment response, and understanding mechanisms of resistance. With the advent of massively parallel DNA and RNA sequencing technologies, it is now possible to computationally predict neoantigens based on patient-specific variant information. However, numerous factors must be considered when prioritizing neoantigens for use in personalized therapies. Complexities such as alternative transcript annotations, various binding, presentation and immunogenicity prediction algorithms, and variable peptide lengths/registers all potentially impact the neoantigen selection process. There has been a rapid development of computational tools that attempt to account for these complexities. While these tools generate numerous algorithmic predictions for neoantigen characterization, results from these pipelines are difficult to navigate and require extensive knowledge of the underlying tools for accurate interpretation. This often leads to over-simplification of pipeline outputs to make them tractable, for example, limiting prediction to a single RNA isoform or only summarizing the top ranked of many possible peptide candidates. In addition to variant detection, gene expression, and predicted peptide binding affinities, recent studies have also demonstrated the importance of mutation location, allele-specific anchor locations, and variation of T-cell response to long versus short peptides. Due to the intricate nature and number of salient neoantigen features, presenting all relevant information to facilitate candidate selection for downstream applications is a difficult challenge that current tools fail to address.

**Results:**

We have created pVACview, the first interactive tool designed to aid in the prioritization and selection of neoantigen candidates for personalized neoantigen therapies including cancer vaccines. pVACview has a user-friendly and intuitive interface where users can upload, explore, select, and export their neoantigen candidates. The tool allows users to visualize candidates at multiple levels of detail including variant, transcript, peptide, and algorithm prediction information.

**Conclusions:**

pVACview will allow researchers to analyze and prioritize neoantigen candidates with greater efficiency and accuracy in basic and translational settings. The application is available as part of the pVACtools software at pvactools.org and as an online server at pvacview.org.

**Supplementary Information:**

The online version contains supplementary material available at 10.1186/s13073-024-01384-7.

## Background

**Fig. 1:**
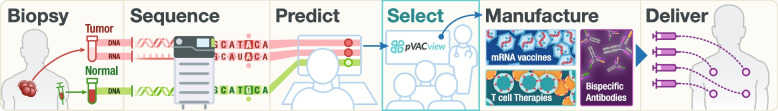
Personalized neoantigen prioritization and therapy development pipeline. The process of developing personalized neoantigen therapy includes six main steps as depicted in this figure. The first step involves patient enrollment and collection of a tumor biopsy and matched normal sample. Next the samples undergo whole genome/exome and RNA sequencing, followed by variant calling to identify somatic variants unique to the cancer, as well as HLA typing. Information regarding the patients’ variants and HLA type are fed to an ensemble of algorithms that predict neoantigen candidates. The candidates are then prioritized based on a multitude of criteria such as binding affinity, presentation, immunogenicity, variant clonality, and variant expression. Finally, the selected candidates are sent to therapy manufacturers (e.g., peptide or nucleic acid cancer vaccines), subjected to safety testing, and ultimately delivered to the patient. pVACview is developed to aid the candidate prioritization and selection step

Neoantigens are unique peptide sequences generated from somatic variants in tumors. These antigens provide an avenue for tumor-specific immune cell recognition and have been found to be important targets for cancer immunotherapies [1–3]. Effective neoantigens, presented by the major histocompatibility complex (MHC) and thus introduced to the patient’s immune system, can prime and activate CD8 + and CD4 + T cells for downstream signaling of cell death. Previous studies have shown that patients with relatively high tumor mutation burden tend to have stronger responses to neoantigen-based immunotherapy treatments [4–6]. With the advent of massively parallel DNA and RNA sequencing technologies, it is now possible to computationally predict neoantigens for experimental studies of T cell biology in cancer or for design of personalized neoantigen therapies based on patient-specific mutations. Examples of such therapies include personalized neoantigen vaccines [2, 7], TCR mimic antibodies [8, 9], personalized adoptive T cell therapies [10, 11], and engineered T cell therapies [12–14]. The process of designing bespoke neoantigen targeting therapies entails sequencing (WGS/WES, RNAseq) of matched tumor-normal samples, somatic variant calling together with germline variant calling and HLA typing, neoantigen prediction, and selection of neoantigen candidates for manufacturing (Fig. 1).

**Fig. 2:**
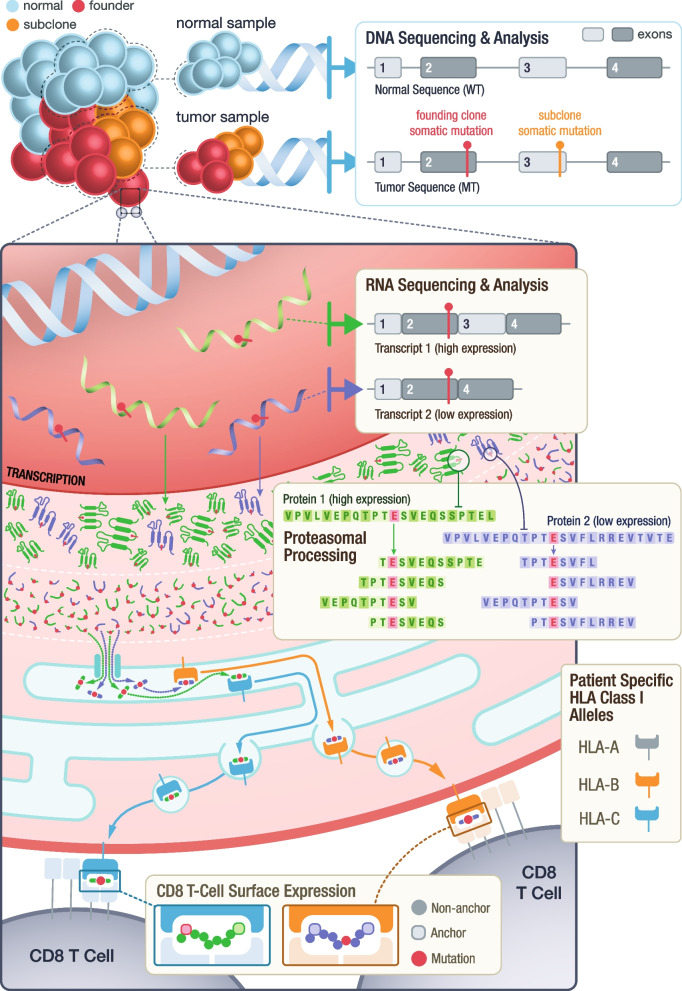
Overall process of neoantigen generation and presentation from tumor specific mutations. Illustration demonstrates the key steps throughout the process of neoantigen generation, processing, binding, transportation, and presentation. This overview highlights examples of criteria examined by pVACview that may be considered during neoantigen prioritization including (1) founding clone versus subclonal tumor status of variants, (2) the impact of different RNA transcript structures on peptide sequence and their varying expression levels, (3) proteasomal processing generating peptides of varying length from different registers, (4) potential for peptide binding to individual patient MHC alleles and the predicted immunogenicity of peptide:MHC complexes, and (5) variant and anchor positions of the neoantigen when presented by MHC to T cells

Numerous aspects of the process of neoantigen generation and presentation must be considered for effective target selection (Fig. 2). These aspects include but are not limited to (1) neoantigen mutation identification and expression, (2) peptide processing and transport, (3) peptide-MHC binding, (4) peptide-MHC stability, and (5) recognition by cytotoxic T cells [15]. Additional considerations relate to manufacturability, which vary by therapeutic platform, and safety considerations including stability of the formulation and potential for off-target effects. There has been a rapid development of computational tools in an attempt to account for these complexities (Additional file 1). Pipelines have been developed to allow researchers to run an ensemble of many tools for individual patients, generating more than 118 features, which include metrics such as algorithmic predictions of binding, allele frequency and expression, similarity to a reference proteome, and others [16–19]. However, the results from these complex pipelines are often overwhelming in number, difficult to navigate, and require extensive knowledge of the underlying tools for accurate interpretation. Though gene expression and predicted peptide binding affinities are common features of most approaches, recent studies have also shown the importance of mutation location, allele-specific anchor locations, the potential impact of multiple class I/II short peptides arising from a single mutation, and the variation of T-cell response to long versus short peptides [20–23]. These additional complexities can be difficult to interrogate directly from computational pipeline outputs, if they are available at all.

With the high cost of personalized neoantigen therapies and difficulties in accurate neoantigen prioritization, it is critical to provide multiple levels of information to support the most effective and efficient neoantigen target selection. As with most personalized treatments, choosing the right approach depends on integrating clinical information and observations with genomic data and algorithmic outputs. Supporting this effectively means moving beyond static reports and building dynamic interfaces that provide layered information in an intuitive manner. To address these concerns, we built a comprehensive visualization tool, pVACview, that takes complex neoantigen candidate information as input, visualizes the output with multiple levels of detail, and exports results and annotations for further review and manufacturing for diverse downstream applications, including personalized neoantigen vaccines. Our previously published cancer immunotherapy software, pVACtools [16], generates numerous features for neoantigen characterization. However, these outputs also require extensive additional analysis due to the intricate nature of neoantigen features. With pVACview, we now provide a complete neoantigen detection and design pipeline. The application is compatible with data from human, mouse, canine, and other species and has been used in the setting of several cancer immunotherapy clinical trials (e.g., NCT04397003, NCT03422094, NCT04015700). It also supports visualization of candidate neoantigens from alternative pipelines such as vaxrank [24] and annotation tools such as NeoFox [17].

## Implementation

pVACview is written in R and is implemented as part of pVACtools, which is a computational toolkit that helps identify and visualize neoantigen candidates [16, 25]. While pVACview can be used as a stand-alone tool (see “Overall architecture of the software implementation”), we recommend using pVACtools to generate the required inputs in order to access the maximum functionality. Code changes are integrated using GitHub pull requests (https://github.com/griffithlab/pVACtools/pulls). Documentation is hosted on Read the Docs (readthedocs.org) and can be viewed at https://pvactools.readthedocs.io/en/latest/pvacview.html.

A demonstration data set is provided and consists of class I and class II neoantigen candidate files generated from the HCC1395 breast cancer cell line and its matched lymphoblastoid cell line HCC1395BL (please refer to data availability section). The tumor and normal datasets were processed using an immunogenomics pipeline written in WDL (immuno.wdl available at https://github.com/wustl-oncology/analysis-wdls). This pipeline accepts raw tumor/normal exome and tumor RNA-seq data in FASTQ or unaligned BAM format and performs alignment, HLA typing, germline variant calling, somatic variant calling, variant phasing, variant annotation, expression analysis, RNA fusion detection, and neoantigen identification. The pipeline also generates the aggregated neoantigen reports and metrics files used as inputs to pVACview. These datasets are available at https://github.com/griffithlab/pVACtools/tree/latestpvactools/tools/pvacview/data.

To acquire pVACtools output (specifically, pVACseq output) for use with pVACview, users can run pVACseq from the command line using variants from their own pipeline (in VCF format), or start with raw sequence data and use an end-to-end pipeline on the cloud by launching our pre-configured workflow on Dockstore (https://dockstore.org/workflows/github.com/griffithlab/analysis-wdls/immuno) via various platforms (e.g., DNAnexus, Terra, eLazi, AnVIL, NHLBI BioData Catalyst). A step-by-step guide for employing the pre-configured immuno workflow to run pVACtools on Terra is available at https://workflow-course.pvactools.org/index.html.

### Overall architecture of the software implementation

pVACview has three modules: (1) main, (2) NeoFox, and (3) custom. The main module supports output from pVACseq while the NeoFox and custom modules support exploration of output from other neoantigen prediction tools. A detailed comparison of neoantigen features provided by pVACseq and several of these alternative prediction tools is provided in Additional file 2.

### pVACview main module

**Fig. 3:**
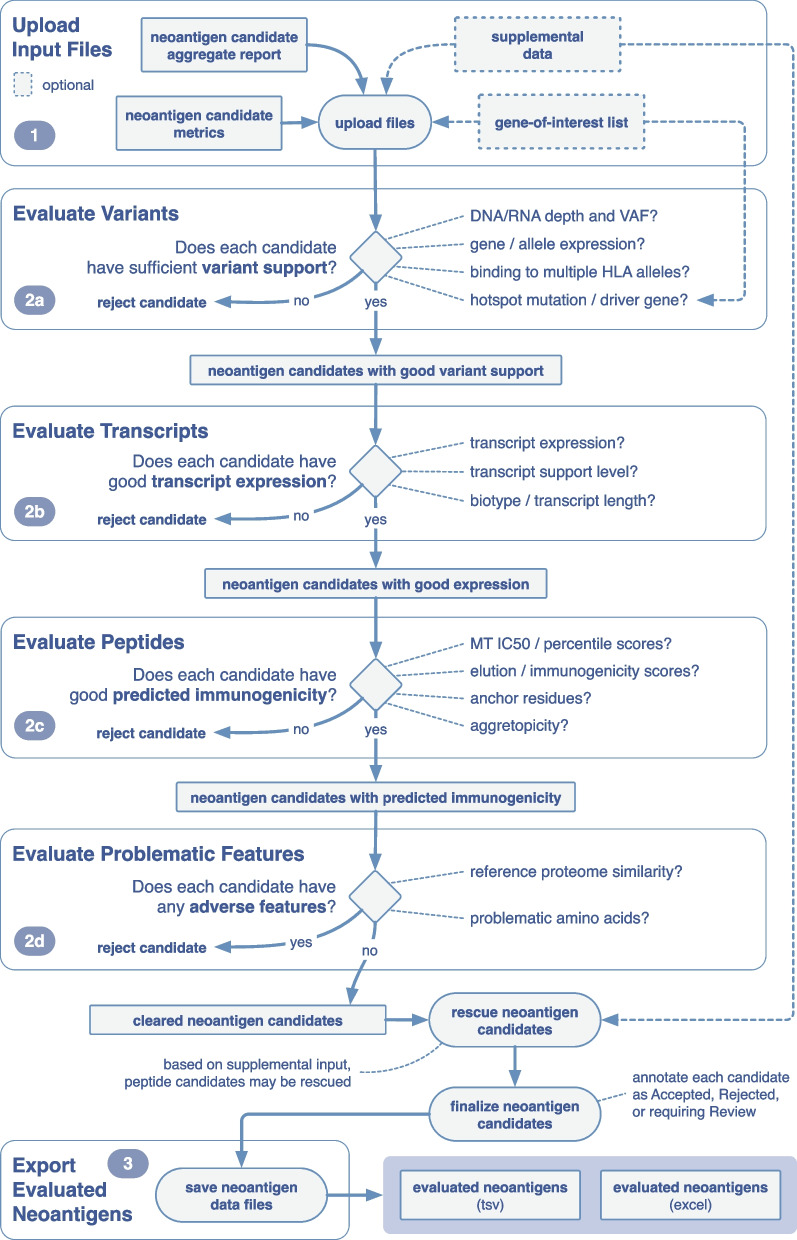
Overview of example workflow for prioritizing neoantigens using pVACview. pVACview can be broken down into three main sections: upload, visualize/explore, and export. When exploring the neoantigen candidates, users are presented with three levels of information: variant, transcript, and peptide. This example workflow guides the user through critical questions that may be considered when prioritizing neoantigen candidates. Each section is organized by the corresponding feature in the pVACview interface

The pVACview main module is split into the following components: user data upload, neoantigen feature visualization and exploration, and export of prioritized neoantigens and associated annotations for downstream applications (Fig. 3). Below, we step through these components in detail. A screenshot and description of each visual element of pVACview can also be found in Additional file 3.

#### Configuration and data import

Generation of the neoantigen candidate input files requires preprocessing using pVACseq starting from patient samples’ variant information (supplied as a VCF file). pVACseq produces neoantigen candidates with numerous features to be considered during prioritization. Two of pVACseq’s output files, an aggregated candidate file (tsv format) and a metrics file (json format), serve as input files to pVACview (Additional file 3: Fig. S1). The aggregated candidate file contains a list of all variants with summary-level information, including the best predicted neoantigen candidate and its overall prediction score, DNA/RNA depth, variant allele frequencies, gene and allele expression, and more. The metrics json file contains extensive additional variant, transcript, peptide, and individual algorithm-level information that is needed for certain features of the pVACview application. For further details, please refer to the online documentation at pvactools.org.

Users have the option to additionally include a tsv file with supplemental candidate information from a different set or class of HLA alleles. This allows users to view basic median binding information of class II results while looking at detailed class I prediction results or vice versa. For users investigating a specific gene set of their own interest, we provide the option of uploading a tsv file where each line contains an individual gene name (e.g., names of known cancer driver genes). These genes, if found in the aggregate report file, will be highlighted in a green box with bold font in the Gene report column of the visualization interface.

#### Neoantigen visualization and exploration

Uploaded neoantigen candidates can be explored and analyzed in several different ways. Users are provided with neoantigen features that are organized into three levels of detail: variant-level, transcript-level, and peptide-level (Fig. 3).

Variant-level information is presented in the main aggregate report table, showcasing the best neoantigen candidate for each variant as well as genomic information (e.g., gene identifier, amino acid change, and position of the variant within the core binding peptide), expression level, DNA/RNA variant allele frequency, median binding prediction scores, percentile ranks, and the total number of peptides beyond the best one that meet specified cutoffs (Additional file 3: Fig. S2). Each variant in the main aggregate report table is assigned to an overall tier based on criteria including binding affinity, expression, transcription support level, clonality, and anchor scenario. By default, the variants in this table are ordered based on their assigned tier.

Once a specific variant is selected, users are provided with a variant and gene info box, which provides further information on the exact genomic location and nucleic acid change (Additional file 3: Fig. S3). We have also included a link to the OpenCRAVAT variant report for the respective variant [26]. This report allows users to explore rich variant information including variant effect annotations, associated cancer types, population allele frequencies, clinical relevance, gene annotation, and pathogenicity predictions.

Additionally, users are provided with individual transcripts containing the variant. The selected variant may occur within multiple transcripts, which may result in distinct neoantigen peptide sequences. Peptides that produce good binding predictions against at least one HLA allele are shown in the transcript table (Additional file 3: Fig. S4). The expression level of each transcript is provided as further guidance when selecting the best neoantigen candidate. In some cases, transcript sequence context impacts the peptide sequence surrounding a variant (e.g., nearby exon–intron boundaries as depicted in Fig. 2). Multiple transcripts that give rise to the exact same list of peptide candidates are grouped into a single transcript set and those that give rise to different peptides are grouped into distinct transcript sets.

Upon selecting a specific transcript set, users are provided with a peptide table (Additional file 3: Fig. S5). The peptide table displays all peptide sequences from the selected transcript that are predicted to be good binders (for at least one HLA allele). Both mutant (MT) and wild type (WT) sequences are shown, along with median binding affinities (if the MT score passed the binding threshold), potential problematic positions for manufacturing, and whether non-specificity of the peptide sequence could indicate potential for autoimmunity or central tolerance [23].

By selecting each pair of MT/WT peptides, users can access (1) plots of the individual IC50 binding affinity predictions of the strong binding MT peptides and their corresponding WT, (2) plots of the individual percentile binding affinity predictions, (3) a binding affinity table with numerical IC50 and percentile rank values across algorithms used, and (4) a table of prediction scores from algorithms trained on mass spectrometry elution data (e.g., BigMHC_EL, MHCFlurryEL, NetMHCPanEL) and immunogenicity data (e.g., BigMHC_IM, DeepImmuno) (Additional file 3: Figs. S6, S7, S8, S9). Note that each peptide may have up to 8 binding algorithm scores for class I alleles (with pVACseq version 3.0 or higher) or up to 4 binding algorithm scores for class II alleles. These views facilitate evaluation of algorithm concordance and integration of predictions pertaining to MHC binding, processing, and immunogenicity.

For each peptide, we also provide users with an allele-specific anchor prediction heatmap, based on computational predictions from our previous work [23]. These predictions are normalized probabilities representing the likelihood of each position of the peptide to participate in anchoring to the HLA allele. The top 15 MT/WT peptide pairs per HLA allele from the peptide table are shown with anchor probabilities overlaid as a heatmap. The anchor probabilities shown are both allele and peptide length specific. In the anchor heatmap view, the mutated amino acids are marked in red and MT/WT pairs are separated using a dotted line (Additional file 3: Fig. S10). The probabilities used for determining allele specific anchors sites are provided along with the actual positions that are considered anchors for each allele-peptide length combination (Additional file 3: Fig. S11). Different anchor scenarios are also depicted to guide users during candidate evaluation (Additional file 3: Fig. S12).

To ensure that the candidate is a non-self peptide, users can also check if the sequence of the peptide candidate matches any sequence found in the reference proteome (Additional file 3: Fig. S13). If the user specifies potential problematic amino acids when running pVACseq, candidates with these problematic amino acids will be flagged by a red box in the “Prob Pos” (Problematic Positions) column of the main aggregate report table (Additional file 3: Fig. S14). One example use of this feature is to flag cysteines (C) as problematic and deprioritize peptides containing them to avoid peptide synthesis and stability issues associated with this amino acid [27].

After consulting the breadth of information displayed in pVACview, users can assign an evaluation to each variant by clicking the appropriate evaluation button in the aggregate report view (Additional file 3: Fig. S15). The number of evaluations performed (accept, reject, review) are tracked in the peptide evaluation overview section. Users may also record a comment for each candidate describing, for example, any notable features, concerns, or special criteria considered to determine the selected evaluation.

If a user has uploaded a tsv file with supplemental candidate information, this data can be viewed in the Additional Data tab (Additional file 3: Fig. S16). This data can, for example, be used to prioritize candidates with poor class I binding affinity but otherwise good metrics. Such candidates may have good class II binding and can be rescued.

#### Export of neoantigen evaluations and final report

When users have either finished evaluating neoantigen candidates or need to pause and would like to save current evaluations, they can export the current main aggregate report using the export page (Additional file 3: Fig. S17). pVACview provides two download file types (tsv and excel). The excel format is user-friendly for downstream visualization and manipulation. However, if the user plans to continue editing the aggregate report and would like to load it back in pVACview with the previous evaluations preloaded, they must use the tsv format. The export feature thus serves as a way to save progress as all evaluations are cleared upon closing or refreshing the pVACview app.

### NeoFox module

#### Data import

pVACview also takes the output of the neoantigen annotation pipeline NeoFox [17] as input. NeoFox output is a tab-separated file, where each row corresponds to one neoantigen candidate. The NeoFox format also optionally supports annotation of each candidate with a patient identifier and gene-level information (gene name, DNA/RNA allele frequencies). The peptide-level information generated by NeoFox is comprehensive and includes scores for ranking peptides based on 16 neoantigen features and prediction algorithms. These features include several that are not otherwise supported by pVACtools directly such as recognition potential, generator rate, PRIME, and HEX [17].

#### Neoantigen visualization and exploration

pVACview provides three panels for NeoFox data exploration. The first panel “Annotated Neoantigen Candidates using NeoFox” will show all neoantigen candidates and their corresponding information from the input. In the second panel “Data Visualization,” users can select up to 6 information categories of the neoantigens to visualize in the form of violin plots. If the user selects a specific peptide in the first tab, the corresponding values of the peptide will be highlighted in red in the plot(s). The third panel “Dynamic Scatter Plot” gives an overview of characteristics of all candidates in the dataset. Users can choose the variables to plot on the x and y axis, as well as the variable which defines the size of the scatter plot. The variables can be transformed and limited in range, if desired. As the user hovers the cursor over any candidate, all information tied to the candidate will be displayed. With these features, users can quickly and interactively narrow down candidates satisfying criteria of interest. A curated subset of NeoFox scores that we believe are particularly useful and/or complementary to that provided by pVACtools are selected by default in the pVACview NeoFox data exploration module. Users can display additional columns by selecting from the “Column visibility” dropdown.

Similar to the main module, users can select an evaluation for each variant by clicking the desired evaluation button in the annotated neoantigen candidates table. The number of evaluations performed (accept, reject, review) are tracked in the “Peptide Evaluation Overview” section on the top left of the page. Users are also able to leave a comment for the selected variant(s) in the section on the top right of the page.

#### Export of neoantigen evaluations and final report

The NeoFox module offers the same export functionalities as the pVACview main module. During export, the selected evaluations and comments are saved to a tsv or excel file alongside the original NeoFox data.

### Custom module

#### Data import

Users can also supply pVACview with any tsv file from any neoantigen prediction algorithm or pipeline. The custom module reads each column in the tsv as a feature and further tailors the view based on user’s selected options in the three following drop-down menus. (1) “Group peptides by” will group peptides together by a user-selected feature. For example, grouping by variant would consolidate all candidate peptides derived from a common variant. (2) “Sort peptides by” will order the candidate peptides by a user-selected feature. For example, a user might order peptides by binding score. (3) "Features to display for each group of peptides" is used to select which features in the dataset will be included in the detailed data section. By default, all features, with exception of the features chosen to group and sort peptide by, will be included. To demonstrate the custom input module, we provide users with example results from other neoantigen prediction pipelines: vaxrank [24], NeoPredPipe [28], and antigen.garnish [29].

#### Neoantigen visualization and exploration

The custom module of pVACview offers three panels for data visualization. The first panel “Overview of Neoantigen Features” displays groups of peptides. For each group, a single representative peptide will be shown. To see and compare the representative peptide with other peptides in the same group, users can click “Investigate” and see all peptides in the second panel—“Detailed Data.” In this second panel, the peptides in the group by default will be sorted by the user-selected feature. The third panel “Dynamic Scatter Plot” allows users to quickly and interactively narrow down candidates satisfying criteria of interest (as described in the “NeoFox module” section above).

Overall, pVACview provides a complex interactive interface to explore many neoantigen features and prioritize neoantigen candidates. A comprehensive analysis of the biological rationale and relative importance of individual features is beyond the scope of this report but several reviews and detailed guidelines have been published [15]. In addition, we provide a list of suggested features and a brief description of their use in candidate prioritization in Table 1. More extensive discussion of many of these features is provided in instructional videos and a comprehensive vignette available in the online documentation (see Availability of data and materials).

**Table 1:** Summary of pVACview features that facilitate neoantigen prioritization

Level	Feature	Suggestion
Variant	RNA Expr: gene expression value for the annotated gene containing the variant RNA VAF: tumor RNA variant allele frequency (VAF) at this position Allele Expr: RNA Expr × RNA VAF RNA Depth: tumor RNA depth at this position DNA VAF: tumor DNA variant allele frequency (VAF) at this position	Prioritize variant(s) with high tumor variant allele fraction (VAF) and allelic expression. Interpretation of tumor VAF first requires estimation of tumor purity which may be guided by the VAF of known tumor drivers Prioritize variants in genes known to be associated with cancer type of the investigated sample (for example, genes listed in Cancer Gene Census)
Transcript	TSL: transcript support level of the transcript coding for the best peptide Biotype: biotype of the transcript coding for the best peptide	Prioritize high-confidence transcripts of level 1 (TSL = 1) Prioritize transcripts(s) with a protein_coding biotype
Peptide	IC50 MT: lowest or median IC50 binding affinity of the best-binding mutant epitope across all prediction algorithms used %ile MT: lowest or median binding affinity percentile rank of the best-binding mutant epitope across all prediction algorithms used (those that provide percentile output) Elution score: likelihood that a peptide will be bound and presented by the MHC molecule, generated by algorithms trained on mass spectrometry data Immunogenicity score: prediction of whether the neoantigen candidate will induce an immune response Ref Match: whether the best peptide is found in the reference proteome (true/false) Prob Pos: positions within the peptide sequence where the amino acid was categorized as problematic for manufacturing purposes	Prioritize peptide(s) with favorable binding affinity to MHC (we recommend binding affinity of 500 nM or less and percentile rank of 2 or less) [30] Prioritize peptides with favorable anchor scenarios (see suggestion by Xia et al. [23]) Prioritize peptides with favorable elution score (elution score from BigMHC_EL, MHCFlurryEL, and NetMHCIIpanEL ranges from 0 to 1, with 1 being the best elution score), with a percentile rank of 2 or less Prioritize peptides with favorable immunogenicity scores (immunogenicity score from DeepImmuno and BigMHC_IM ranges from 0 to 1, with 1 being the best elution score) Exclude peptides with a reference match in the proteome Exclude peptides with problematic amino acids

## Results and discussion

Multiple tools and workflows have been created for neoantigen characterization, both for studies of tumor T cell biology and the development of neoantigen-based therapies. Some existing tools that address individual factors for prediction of neoantigens do include visualization components (such as netMHC for binding predictions). Pipelines such as pVACtools and NeoFox combine multiple algorithms for detecting, characterizing, and prioritizing neoantigens from various sources. However, these pipelines do not facilitate visual exploration of the results, instead producing a static neoantigen report that often attempts to provide only a single “best” neoantigen for each variant based on simple criteria such as binding affinity predictions. During this process, these reports over-simplify the outputs to make them tractable, and thus the results are fraught with assumptions about what “best” means. This reduces the ability to effectively prioritize neoantigen candidates. A more nuanced approach that allows consideration of multiple contextually relevant features is preferable. We therefore believe that an interactive neoantigen visualization tool, customized to this specific application, is needed. Two existing tools, NeoPredViz [28] and LENS [31], do offer visualization of their results. However, pVACview remains distinct in its ability to present a diversity of variant, transcript, peptide, and algorithm data together in a simple, integrated view. As a dynamic interactive visualization interface, pVACview overcomes many limitations of tabular reports, allowing the user to consider neoantigens in the context of transcript expression, tumor clonality, multiple registers (peptides of the same length where the variant is at different positions), peptide lengths, alternative transcript isoforms, an ensemble of predictive binding algorithms, HLA specific anchor information, and much more.

pVACview helps users to address many complexities of neoantigen interpretation and prioritization that are difficult to achieve with tabular reports. While there has been a rapid development of sequencing technologies, bulk tumor tissue samples undergoing sequencing are often subject to purity issues. Additionally, intratumoral heterogeneity presents a considerable challenge to cancer therapies, making it critical to distinguish variants from the founding clone from those that are subclonal. Neoantigens arise from tumor-specific genomic variations and each variant can have multiple transcripts encompassing the variant location. Thus, not only should transcript-specific expression level be taken into account, transcripts resulting from different splicing patterns may also have considerable impact on the exact neoantigen peptide sequence. Once the correct sequence surrounding a variant from an expressed transcript is identified, neoantigen candidates can be extracted by looking at different registers and different lengths of peptides containing the amino acid modification. Even for the simplest form of neoantigen sources, single nucleotide variants, when all registers, lengths (8-, 9-, 10-, 11-mer) and algorithms (13 for class I) in pVACtools are used, and assuming an individual with 6 distinct class I alleles, the result is 38 distinct short peptides and 2964 peptide MHC predictions. Neoantigens arising from frameshift variants can produce an even larger number of neoantigen candidates that vary in sequence and variant position, with each peptide having a different set of prediction scores. Filtering by binding affinity thresholds or other criteria can reduce the complexity of this result to a degree but it often remains unwieldy. The detailed information provided by pVACview can effectively help users address this complexity.

Furthermore, pVACview’s drill down approach to information display helps researchers intuitively integrate variant clonality, class I and II binding predictions, competing binding prediction algorithms, binding metrics, and mutation positional information for each candidate neoantigen peptide. Expressed neoantigens of different lengths and registers from a founding clone of the tumor can potentially bind (or not bind) to either class I or class II HLA alleles, either through endogenous or exogenous pathways respectively on either the tumor cell or an antigen presenting cell. In addition to having up to 6 different class I HLA alleles, each patient may have up to 12 different class II alleles (and dimer combinations of these). When evaluating neoantigen candidates in terms of binding predictions, researchers may take into account how well neoantigens are potentially binding to each patient-specific HLA allele and may also want to consider how many different HLA alleles it can bind robustly [32, 33]. pVACview provides the ability to consider the diversity of peptides arising from each variant and how they relate to predicted binding by multiple alleles. Another approach to accounting for multiplicity of presentation is the Patient Harmonic-mean Best Rank (PHBR) score [34, 35], conveniently provided by NeoFox and supported in pVACview. As described, for each neoantigen, there are numerous prediction algorithm results available. How the scores are distributed across different algorithms and whether the IC50 binding prediction or percentile rank value should be utilized are all important aspects that require careful evaluation. If a neoantigen is expressed and predicted to bind well to an HLA allele, researchers should further consider the anchor and mutation locations of the peptide-MHC pairing. A subset of peptide positions are primarily presented to the T-cell receptor for recognition, while others are primarily responsible for anchoring to the MHC (though neither role is exclusive). Whether the mutation lies in an anchor region and how well the WT peptide binds to the MHC create different scenarios that can influence whether a neoantigen remains a good candidate [21, 23].

Finally, in the setting of clinical trials, additional details influence the priority of neoantigens. Tumor samples are first collected from patients and put through a series of genomic analysis pipelines, including DNA and RNA sequencing, variant detection, and expression estimation. Pipelines such as pVACtools then take these results and identify possible neoantigen candidates. Throughout this process, problems such as low tumor purity, contamination, and insufficient or excessive neoantigen candidates may arise. pVACview allows users to promptly adjust tiering of candidates based on tumor purity and expression levels. It also highlights the specific failing criteria, providing users the option to further explore criteria such as how to define anchor positions, with the flexibility of rescuing candidates for samples with insufficient candidates for downstream applications such as neoantigen vaccines. For cases where an excessive number of neoantigen candidates exist, pVACview effectively prioritizes candidates (based on calculated tiering, allele expression, and average mutant peptide binding affinity) while simultaneously allowing users to sort and annotate candidates based on features of their own choosing.

All the aspects described above are potentially critical in order to infer whether the presenting peptide sequence can successfully induce an immune response. pVACview was designed to present this complex information to researchers in an intuitive manner and aid in the prioritization and selection of neoantigen candidates for personalized cancer vaccines or other therapeutic and research applications. Basic and translational researchers can use pVACview to visualize neoantigen candidates along with detailed supporting information including that of the genomic variant, transcripts affected by the variant, and good-binding peptides predicted from the respective transcripts.

## Conclusions

Accurate neoantigen prediction is critical to cancer immunotherapy treatments and several tools have been built to account for individual aspects throughout this process. However, these tools lack methods for integration and visualization, making it challenging for researchers to efficiently explore the many molecular and algorithmic features relevant to neoantigens, such as variant-, transcript-, and peptide-level information. pVACview integrates multiple levels of information into a visualization tool, allowing users to analyze each candidate in detail for optimal decision-making. This tool has been tested and used in clinical trials and research projects involving human, mouse, and canine model systems. We hope by using pVACview, researchers can analyze and prioritize neoantigen candidates with greater efficiency and accuracy. The application is available as part of the pVACtools pipeline and as an online web tool hosted on the Google Cloud Platform at www.pvacview.org.

## Supplementary Information

Additional file 1. Neoantigen prediction pipelines. Comparison of neoantigen prediction pipelines.

Additional file 2. pVACview inputs. Comparison of variant-, transcript-, and peptide-level information in the outputs of five neoantigen prediction pipelines: pVACtools, NeoFox, vaxrank, antigen.garnish, NeoPredPipe. These prediction pipeline outputs serve as examples of pVACview inputs.

Additional file 3. pVACview main module feature overview. Screenshots and descriptions of all features in pVACview main module.

## Data Availability

Availability and requirements The FASTQ and BAM files for HCC1395 and HCC1395BL demonstration data can be found at NCBI BioProject accession number: PRJNA201238 (SRA accession number: SRX285805, SRX285804, SRX278523, SRX278522, SRX278521, SRX278520, SRX278519, SRX278518, SRX278517) (https://www.ncbi.nlm.nih.gov/bioproject/201238) [36]. The pVACview codebase is hosted publicly as part of the pVACtools project on GitHub at https://github.com/griffithlab/pVACtools. User documentation (including a vignette for using pVACview to evaluate neoantigen candidates) is available at pvactools.org. A “Tutorials” tab, which explains key functionalities, is also included on the pvacview.org landing page. This project is licensed under the BSD 3-Clause Clear License (https://github.com/griffithlab/pVACtools/blob/master/LICENSE). pVACtools has been packaged and uploaded to PyPI under the “pvactools” package name (https://pypi.org/project/pvactools/) and can be installed on Linux or Mac systems by running the “pip install pvactools” command. Installation requires R and a Python 3.7 or higher environment. Versioned Docker images including all dependencies are available on DockerHub (https://hub.docker.com/r/griffithlab/pvactools/). Releases are also made available on GitHub (https://github.com/griffithlab/pVACtools/releases). Video demonstration We have created a series of demonstration videos that walk through the basic steps of using pVACview using neoantigen candidate files generated from the HCC1395 dataset as input. This shows the full process of launching the application, uploading datasets, exploring neoantigen information, adding comments and marking evaluations, and exporting the data for further usage. Additional videos describing modules customized to support NeoFox results or custom results from any neoantigen prediction pipeline are also featured. The pVACview tutorial playlist is available at https://www.youtube.com/playlist?list=PLQJ7idhjxknRUMcnqjmhoM1t31CYmmNI8.

## Abbreviations

MT: Mutant
WT: Wild type
HLA: Human leukocyte antigen
MHC: Major histocompatibility complex
VAF: Variant allele frequency
VCF: Variant Call Format
