# Supplementary material for: pVACview: an interactive visualization tool for efficient neoantigen prioritization and selection
Source: Genome Med. 2024 Nov 14;16:132. doi: 10.1186/s13073-024-01384-7 (PMC11562694; doi:10.1186/s13073-024-01384-7)
Supplement: Supplementary file 1 — Additional file 1. Neoantigen prediction pipelines. Comparison of neoantigen prediction pipelines. [file 13073_2024_1384_MOESM1_ESM.pdf]

Additional file 1: Neoantigen Prediction Pipelines

Tool Name	Language	Has Visualization	Initial tool publication date	Last Changes on Github (as of May 2024)	Citations (as of May 2024)	Does this tool provide information for multiple peptides derived from 1 individual variant?	Binding prediction algorithms used	Github Repo/Documentation	Best Docker
pVACtools	Python	Yes (pVACview)	Jan 2016 (Updated version: March 2020)	May 2024	406	Yes	NetMHCpan, NetMHC, NetMHCcons, PickPocket, SMM, SMMPMBEC, MHCflurry, MHCnuggets, NetMHCIIpan, SMMalign, NNalign	https://github.com/griffithlab/pVACtools	https://hub.docker.com/r/griffithlab/pvactools
Epi-Seq	Java	No	Oct 2014	N/A (Software version available from Mar 2013)	431	No	netMHC	https://dna.engr.uconn.edu/?page_id=470#:~:text=Epi%2DSeq%20is%20a%20multi,predicted%20tumor%2Dspecific%20expressed%20epitopes.	dockerfile not found
MuPeXi	Python	No	Apr 2017	Feb 2019	136	Yes	netMHCpan	https://github.com/ambj/MuPeXI	https://hub.docker.com/r/openvax/mupexi-grch38
Antigen.garnish	R	No	October 2019	July 2022	119	Yes	NetMHC, NetMHCpan, NetMHCII, NetMHCIIpan	https://github.com/andrewrech/antigen.garnish	https://hub.docker.com/r/andrewrech/antigen.garnish
NeoPredPipe	Python 2.7+	Yes (NeoPredViz)	May 2019	Sep 2023	95	No	netMHCpan, netMHCpanII	https://github.com/MathOnco/NeoPredPipe	https://hub.docker.com/r/eszterlakatos/neopredpipe
NeoFlow	Nextflow, Python, R	No	Apr 2020	July 2023	70	Yes	netMHCpan	https://github.com/bzhanglab/neoflow	https://hub.docker.com/r/bzhanglab/neoflow
TSNAd	Python, Perl	No	Apr 2017 (Updated version: Aug 2021)	Aug 2021	79	Yes	DeepHLApan	https://github.com/jiujiezz/tsnad	https://hub.docker.com/r/biopharm/tsnad
CloudNeo	CWL	No	Oct 2017	Apr 2019	65	Yes, but user would need to extract information and summarize themselves	netMHC, netMHCpan	https://github.com/TheJacksonLaboratory/CloudNeo	dockerfile not found
Epidisco	OCaml (ketrew workflows)	No	Nov 2017 (preprint dedicated to tool), Jan 2018 (Paper mentioned pipeline. Tool has no official publication)	Nov 2017	62	Yes, if providing raw vaxrank output	Algorithms utilized by Vaxrank	https://github.com/hammerlab/epidisco	https://hub.docker.com/r/hammerlab/epidisco-web
Fred2	Python 2.7	No	Jul 2016	Mar 2020	37	Yes	SYFPEITHI, BIMAS, SVMHC, ARB, SMM, SMMPMBEC, Epidemix, Comblib Sidney 2008, PickPocket, NetMHC, NetMHCpan, HAMMER, TEPITOPEpan, NetMHCII, NetMHCIIpan, UniTope, NetCTLpan	https://github.com/FRED-2/Fred2/	https://hub.docker.com/r/fred2/optitype
ptuneos	Python	No	Oct 2019	Nov 2021	36	Yes	netMHCpan	https://github.com/bm2-lab/pTuneos	https://hub.docker.com/r/bm2lab/ptuneos
Vaxrank/OpenVax	Python	No	October 2018 (bioRxiv), Mar 2020 (publication)	Mar 2024	22	multiple peptides from one variant are included if passing all criteria	netMHC, netMHCpan, netMHCcons, MHCflurry, netMHCpanII	https://github.com/openvax/vaxrank	https://hub.docker.com/r/ngebremedhin/vaxrank
NeoFuse	Python, R, Shell	No	Apr 2020	Aug 2022	29	Yes	MHCflurry, netMHCpan	https://github.com/icbi-lab/NeoFuse (https://icbi.i-med.ac.at/software/NeoFuse/NeoFuse.shtml)	https://hub.docker.com/r/icbi/neofuse
ProTECT	Python	No	Nov 2020	May 2018	16	No	IEDB tool suite (netMHCcons, SMMalign, PickPocket etc.)	https://github.com/BD2KGenomics/protect (https://github.com/BD2KGenomics/protect/blob/master/MANUAL.md)	No docker image for overall pipeline (https://hub.docker.com/u/aarjunrao/)
Neoantimon	R	No	Sep 2020	Sep 2023	8	Yes	netMHCpan, MHCflurry, netMHCIIpan	https://github.com/hase62/Neoantimon	dockerfile not found
NeoFox	Python	No	Nov 2021	Oct 2023	10	No	netMHCpan, netMHCpanII, MixMHCpred, MixMHCpred2, Vaxrank, PRIME	https://github.com/TRON-Bioinformatics/neofox	Need to build your personal docker with provided instructions
NextNEOpi	Nextflow, R, Python, java, singularity	Can use pVACview if updated pVACseq 3.0	Feb 2022	Sep 2013	22	Uses pVACtools which does provide peptide level information	netMHCpan, netMHCIIpan, mhcflurry, mixMHC2pred	https://github.com/icbi-lab/nextNEOpi	dockerfile not found
LENS	Nextflow	Yes (LENS Viz)	June 2023	Sep 2022 (Gitlab, LENS Viz)	9	Yes	NetMHCpan-4.1b	https://gitlab.com/landscape-of-effective-neoantigens-software (https://gitlab.com/landscape-of-effective-neoantigens-software/nextflow/modules/tools/lens/-/wikis/home)	Need to build your personal docker with provided instructions
ScanNeo	Python, snakemake	No	Mar 2019 (updated version: Nov 2023)	August 2022	39	Yes	NetMHC, NetMHCpan	https://github.com/ylab-hi/ScanNeo	https://hub.docker.com/r/yanglabinfo/scanneo2-scanexitron
TIminer	Python	No	Oct 2017	N/A (no github)	90	Yes	NetMHCpan	https://icbi.i-med.ac.at/software/timiner/doc/	https://hub.docker.com/r/icbi/timiner
TruNeo	Python, Perl, R	No	Nov 2020	Nov 2020	14	Yes	NetMHCpan	https://github.com/yucebio/TruNeo	dockerfile not found
ProGeo-Neo	Python (V2.7), Perl and Java	No	Apr 2020 (updated version: Apr 2022)	Apr 2022	22	Yes	NetMHCpan	https://github.com/kbvstmd/ProGeo-neo	dockerfile not found
nfcore-epitopeprediction	Nextflow	No	Feb 2020 (paper is for all 30+ pipelines in nfcore)	May 2024	674	Yes	syfpeithi, mhcflurry, mhcnuggets-class-1, mhcnuggets-class-2, netmhcpan, netmhcpan, netmhc, netmhciipan	https://github.com/nf-core/epitopeprediction?tab=readme-ov-file	https://hub.docker.com/r/nfcore/epitopeprediction
Neoepiscope	Python	No	Feb 2020	Aug 2022	25	Yes	mhcnuggets, mhcflurry, netMHCpan, netMHCIIpan	https://github.com/pdxgx/neoepiscope	Need to build your dockerfile
Neopepsee	Java	No	Jan 2020	N/A (no github found)	133	Yes	NetCTLpan, IEDB-Peptide binding to MHC class I molecules, IEDB-T cell class I pMHC immunogenicity predictor	https://sourceforge.net/p/neopepsee/wiki/Home/	dockerfile not found
DeepHLAPan	Perl, Python, bash	No	Nov 2019	Jul 2022	95	user provides peptide level information	DeepHLAPan	https://github.com/jiujiezz/deephlapan	https://hub.docker.com/r/biopharm/deephlapan
