# Supplementary material for: pVACview: an interactive visualization tool for efficient neoantigen prioritization and selection
Source: Genome Med. 2024 Nov 14;16:132. doi: 10.1186/s13073-024-01384-7 (PMC11562694; doi:10.1186/s13073-024-01384-7)
Supplement: Supplementary file 2 — Additional file 2. pVACview inputs. Comparison of variant-, transcript-, and peptide-level information in the outputs of five neoantigen prediction pipelines: pVACtools, NeoFox, vaxrank, antigen.garnish, NeoPredPipe. These prediction pipeline outputs serve as examples of pVACview inputs. [file 13073_2024_1384_MOESM2_ESM.pdf]

Additional file 2: pVACview inputs

Tool/Package Name		pVACtools	NeoFox	Vaxrank	Antigen.garnish	NeoPredPipe
**Variant Level Inputs**	Genomic Coordinates					
	Mutation Position					
	Gene Name					
	Amino Acid Change					
	DNA Depth					
	DNA VAF					
	Gene Expression					
	RNA VAF			Provides "Variant allele RNA read count"		
	RNA Depth					
**Transcript Level Inputs**	Transcript Expression					
	Transcript Support Level					
	Biotype					
	Length					
	Location of variant in transcript					
**Peptide Level Inputs**	Minimum Peptide Sequence and matched HLA					
	Peptide sequences (from different registers of same variant)					
	Peptides from different transcripts				The tool will only predict peptides derived from user-input transcript.	
	Predicted Binding Affinities					
	Multiple prediction algorithms			User needs to compile from multiple output files (each corresponding to one algorithm)		
	Class I and Class II prediction algorithms					
	Multiple binding measurements (e.g. IC50, precentile) available					
	Algorithms with different training data (e.g. in vitro binding, elution data)					Uses netMHCpan-4.1
	Binding results for both wildtype and mutant peptides					
	Binding results available for multiple HLA alleles (not solely best binding HLA allele)					

Generated by tool with input data (or part of required input)	
Can be added by user as annotation but can't be generated by tool	
Does not exist in output file	
