# Supplementary material for: pVACview: an interactive visualization tool for efficient neoantigen prioritization and selection
Source: Genome Med. 2024 Nov 14;16:132. doi: 10.1186/s13073-024-01384-7 (PMC11562694; doi:10.1186/s13073-024-01384-7)

Supplementary Figures

The pVACview main module is split into the following components: (1) user data upload, (2) neoantigen feature visualization and exploration, and (3) export of prioritized neoantigens and associated annotations for downstream applications. Users are provided with neoantigen features that are organized into three levels of detail: variant-level, transcript-level, and peptide-level. The following figures provide screenshots and descriptions of pVACview main module features.

List of Supplementary Figures

Fig. S1. Upload input files
Fig. S2. Visualize and explore - Evaluate and compare variants
Fig. S3. Visualize and explore - Evaluate individual variant details: DNA/RNA VAF, gene expression and genomic variant coordinates
Fig. S4. Visualize and explore - Evaluate and compare alternative transcripts: transcript expression, transcript support level, biotype and transcript length
Fig. S5. Visualize and explore - Evaluate and compare peptides arising from a single variant and transcript set
Fig. S6. Visualize and explore - Evaluate an individual peptide: IC50 binding affinity violin plot
Fig. S7. Visualize and explore - Evaluate an individual peptide: percentile rank (%ile) violin plot
Fig. S8. Visualize and explore - Evaluate an individual peptide: IC50 binding affinity and percentile rank
Fig. S9. Visualize and explore - Evaluate an individual peptide: elution and immunogenicity
Fig. S10. Visualize and explore - Evaluate an individual peptide: anchor residue (heatmap)
Fig. S11. Visualize and explore - Evaluate an individual peptide: anchor residue (tables)
Fig. S12. Visualize and explore - Evaluate individual peptide: anchor scenarios
Fig. S13. Visualize and explore - Evaluate problematic features: reference proteome similarity
Fig. S14. Visualize and explore - Evaluate problematic features: problematic amino acids
Fig. S15. Visualize and explore - Capture evaluation status
Fig. S16. Visualize and explore - Rescuing poor candidate
Fig. S17. Export evaluated neoantigens

Fig. S1. Upload input files
The pVACview main module has two required input files: <sample_name>.all_epitopes.aggregated.tsv and <sample_name>.all_epitopes.aggregated.metrics.json, both of which are output files from the pVACseq pipeline. The aggregated tsv file provides a list of neoantigen producing variants, features of each variant, the best predicted epitope, binding affinity scores and percentile ranks. The metrics json file contains additional transcript and peptide level information that is needed for certain features of the pVACview application. Users can also upload an additional all_epitope.aggregated.tsv file, which is useful in cases where users are visualizing Class I prediction data but would like to have a general idea of the variant's Class II prediction performance or vice versa. Users also have the opportunity to upload a gene-of-interest tsv file, where each individual line consists of one gene name (e.g. cancer driver genes). If matched in the aggregate report, the gene name will be highlighted using bold font and a green box around the cell.

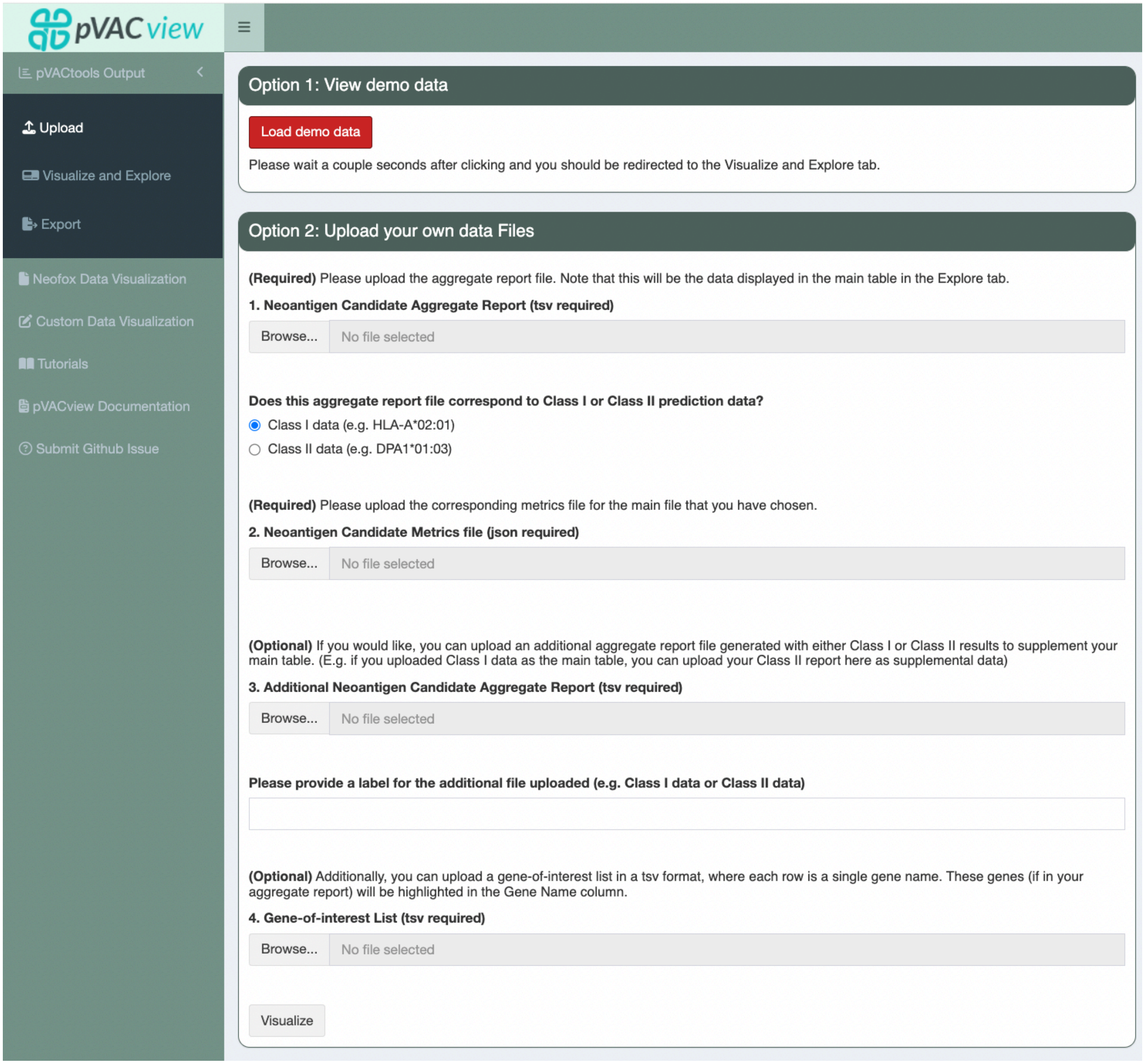

Fig. S2. Visualize and explore - Evaluate and compare variants

Variant-level information is presented in the main aggregate report table, showcasing the best neoantigen candidate for each variant as well as genomic information (e.g. gene identifier, amino acid change and position of the variant within the core binding peptide), expression level, DNA and RNA variant allele frequency (VAF), median binding prediction scores and percentiles, the total number of peptides that meet specified cutoffs, etc. Driver genes provided in the user-provided gene of interest list are framed in green boxes (ARID1B and MSH6 in this example). Further details on each of the features depicted below can be found at pvactools.org and within the pVACview interface itself (Tutorials and Documentation sections).

Aggregate Report of Best Candidates by Variant

Search:

	Gene	AA Change	Best Peptide	TSL	Allele	Pos	Prob Pos	Num Passing Peptides	IC50 MT	IC50 WT	%ile MT	%ile WT	RNA Expr	RNA VAF	Allele Expr	RNA Depth	DNA VAF	Tier	Ref Match	Acpt	Rej	Rev
1	ADAR	E806V	AERMGFTVV	1	HLA-B*45:01	8	None	5	76.11	61.796	0.1	0.125	131.835	0.348	45.879	1233	0.302	Pass	False			
2	KIF1C	S433F	TEFQIGPEEA	1	HLA-B*45:01	3	None	1	152.1	166.310	0.35	0.473	121.453	0.297	36.072	1679	0.316	Pass	False			
3	OSTC	F9L	YRVPLLVL	1	HLA-C*06:02	5	None	1	282.169	272.915	0.232	0.202	173.877	0.486	84.504	1028	0.462	Pass	False			
4	SURF1	N89K	RRKWKLKLI	1	HLA-C*06:02	7	None	2	212.835	161.921	0.451	0.332	46.709	0.762	35.592	563	0.700	Pass	False			
5	**ARID1B**	G910A	SPGGQMHAA	1	HLA-B*82:02	9	None	1	345.587	2240.610	0.61	2.500	39.757	1.000	39.757	163	1.000	Pass	False			
6	HSPA4	L751P	NPQNKQSL	1	HLA-B*82:02	2	None	1	454.22	19898.660	0.49	14.000	114.662	0.996	114.203	1368	1.000	Pass	False			
7	**MSH6**	D1255N	VENYSQNVA	1	HLA-B*45:01	3	None	4	66.515	332.805	0.254	0.665	48.832	0.338	16.505	352	0.318	Pass	False			
8	RPRD1A	Q21H	SELSNSQHSV	1	HLA-B*45:01	8	None	1	368.845	354.640	0.43	0.415	57.249	0.500	28.625	602	0.417	Pass	False			
9	MAU2	S111R	VKFEAARLL	1	HLA-C*06:02	7	None	2	212.075	484.278	0.424	1.251	34.844	0.437	15.227	238	0.345	Pass	False			
10	ASMTL	T445M	AMAFNLSRF	1	HLA-A*29:02	2	None	1	618.761	2037.415	1.579	2.250	74.609	0.460	34.320	804	0.467	Pass	False			

Showing 1 to 10 of 321 entries Show 10 entries

Previous 1 2 3 4 5 … 33 Next

Currently investigating row:

1

Fig. S3. Visualize and explore - Evaluate individual variant details: DNA/RNA VAF, gene expression and genomic variant coordinates

Once a specific variant row is selected in the aggregate report table, users are provided with a ‘Variant and Gene info’ box, which provides further information on the precise genomic location and nucleic acid change, as well as a link to an OpenCRAVAT variant report for the respective variant. The ‘Variant and Gene info’ box is located in the ‘Variant Information’ tab.

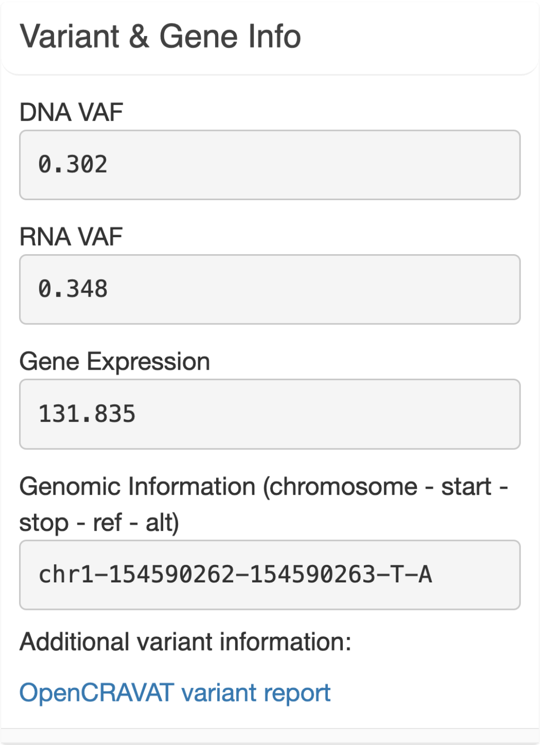

Fig. S4. Visualize and explore - Evaluate and compare alternative transcripts: transcript expression, transcript support level, biotype and transcript length

A) Multiple transcripts that give rise to the exact same list of peptide candidates are grouped into a single transcript set. B) Once a transcript set is selected, users are provided with information on each individual transcript containing the variant. Transcript specific expression estimates, transcript support level (TSL) and biotype can be used to identify a suitable reference transcript sequence for extraction of long peptide sequences that contain a neoantigen. When multiple transcript options are available within a transcript set, the "top" transcript (highlighted in green) is selected using the TSL, biotype and transcript length information.

A. Transcript set selection

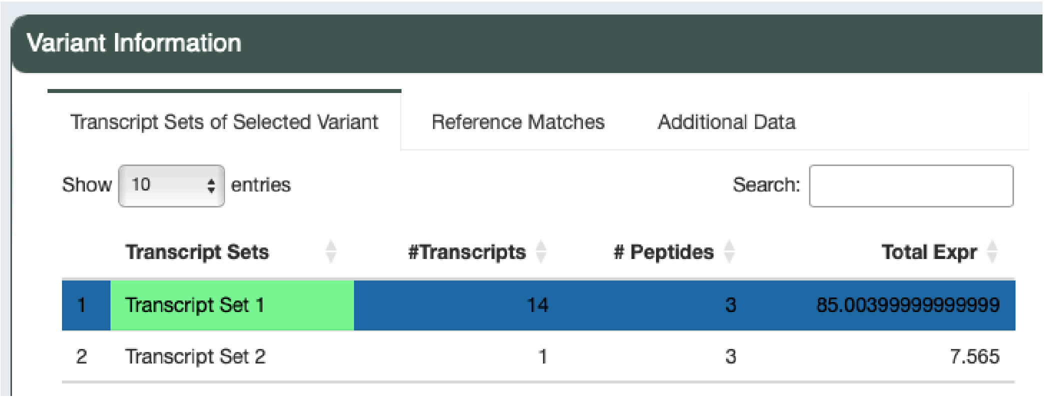

Variant Information

Transcript Sets of Selected Variant | Reference Matches | Additional Data

Show 10 entries — Search:

	Transcript Sets	#Transcripts	# Peptides	Total Expr
1	Transcript Set 1	14	3	85.00399999999999
2	Transcript Set 2	1	3	7.565

B. Transcripts in selected set

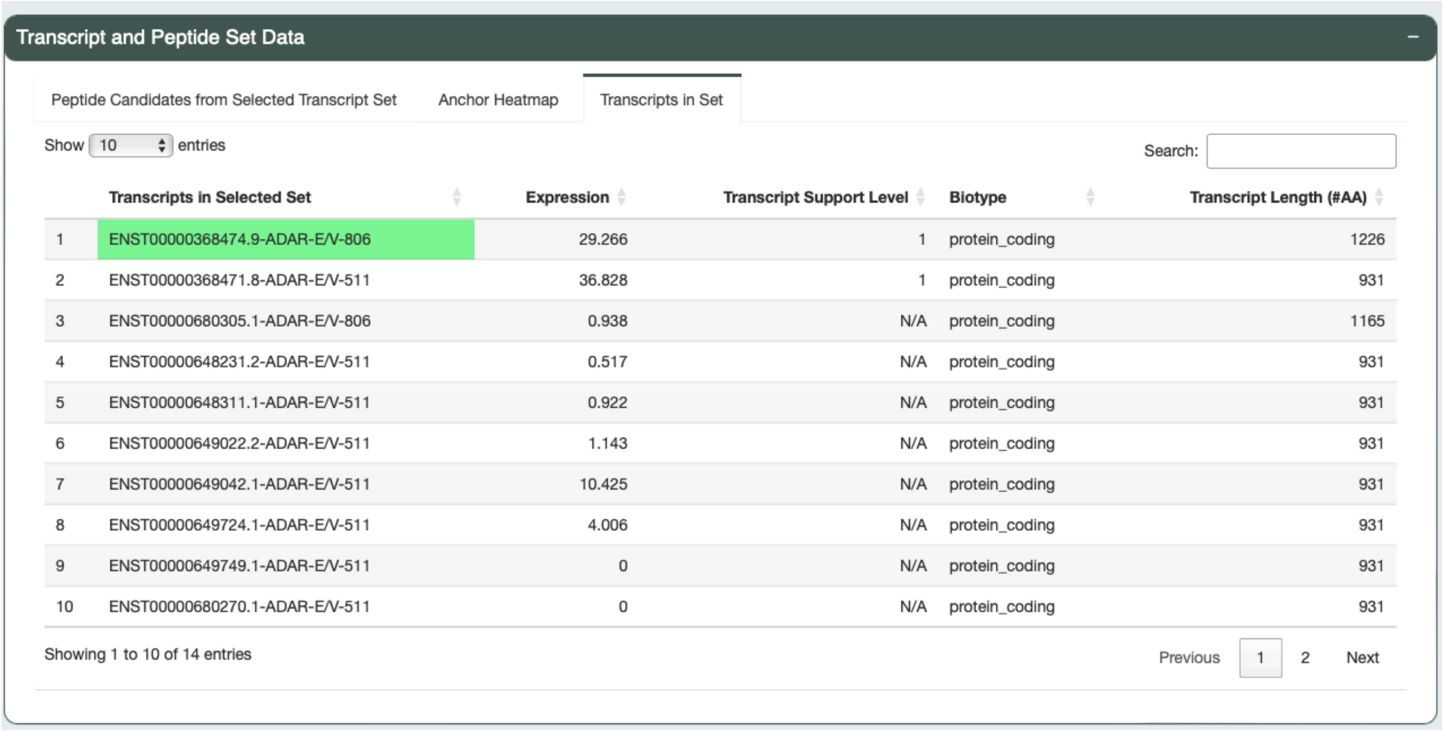

Transcript and Peptide Set Data

Peptide Candidates from Selected Transcript Set | Anchor Heatmap | Transcripts in Set

Show 10 entries — Search:

	Transcripts in Selected Set	Expression	Transcript Support Level	Biotype	Transcript Length (#AA)
1	ENST00000368474.9-ADAR-E/V-806	29.266	1	protein_coding	1226
2	ENST00000368471.8-ADAR-E/V-511	36.828	1	protein_coding	931
3	ENST00000680305.1-ADAR-E/V-806	0.938	N/A	protein_coding	1165
4	ENST00000648231.2-ADAR-E/V-511	0.517	N/A	protein_coding	931
5	ENST00000648311.1-ADAR-E/V-511	0.922	N/A	protein_coding	931
6	ENST00000649022.2-ADAR-E/V-511	1.143	N/A	protein_coding	931
7	ENST00000649042.1-ADAR-E/V-511	10.425	N/A	protein_coding	931
8	ENST00000649724.1-ADAR-E/V-511	4.006	N/A	protein_coding	931
9	ENST00000649749.1-ADAR-E/V-511	0	N/A	protein_coding	931
10	ENST00000680270.1-ADAR-E/V-511	0	N/A	protein_coding	931

Showing 1 to 10 of 14 entries — Previous 1 2 Next

Fig. S5. Visualize and explore - Evaluate and compare peptides arising from a single variant and transcript set

The “Peptide Candidates from Selected Transcript Set” table provides details for peptides arising from the selected transcript set (i.e. for cases where multiple alternative transcripts give rise to the exact same list of peptide candidates). For each peptide, the predicted per MHC allele IC50 binding affinity, mutation position, problematic positions and anchor residue fail status is provided. Each row corresponds to either the mutant peptide or the corresponding wild type peptide sequence, indicated as “MT” and “WT” respectively. Users are able to select MT/WT peptides for further information. The “top” peptide (highlighted in green) is identified by eliminating any problematic peptides and choosing from the remaining peptides the one with the strongest binding score.

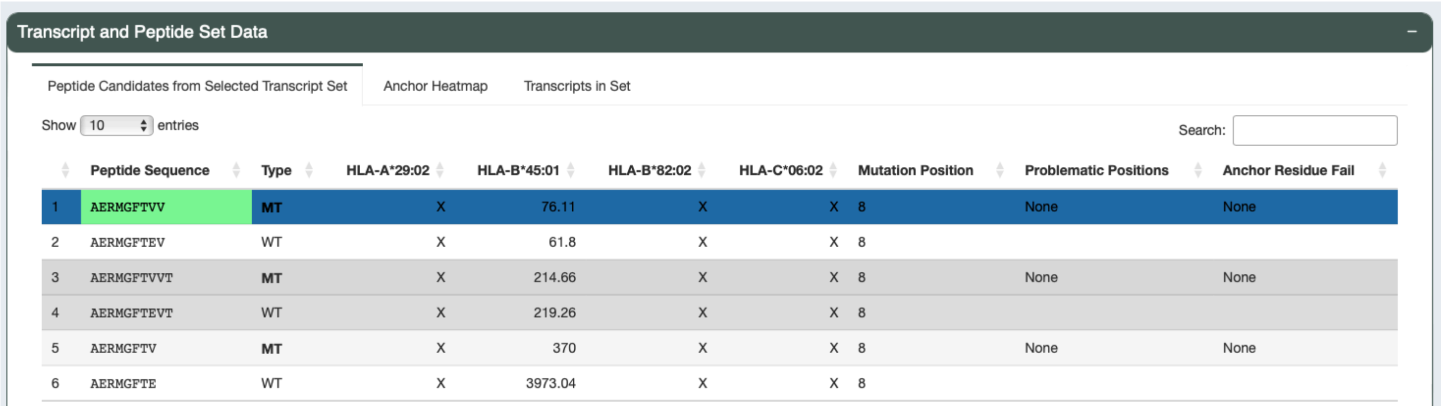
Transcript and Peptide Set Data

Peptide Candidates from Selected Transcript Set | Anchor Heatmap | Transcripts in Set

Show 10 entries

Search:

	Peptide Sequence	Type	HLA-A*29:02	HLA-B*45:01	HLA-B*82:02	HLA-C*06:02	Mutation Position	Problematic Positions	Anchor Residue Fail
1	AERMGFTVV	**MT**	X	76.11	X	X	8	None	None
2	AERMGFTEV	WT	X	61.8	X	X	8		
3	AERMGFTVVT	**MT**	X	214.66	X	X	8	None	None
4	AERMGFTEVT	WT	X	219.26	X	X	8		
5	AERMGFTV	**MT**	X	370	X	X	8	None	None
6	AERMGFTE	WT	X	3973.04	X	X	8		

Fig. S6. Visualize and explore - Evaluate an individual peptide: IC50 binding affinity violin plot
After selecting a single mutant peptide, the IC50 Plot tab shows violin plots of IC50 binding affinity predictions from individual algorithms for the MT (mutant) and matched WT (wildtype) peptides for each HLA allele meeting a binding affinity threshold.

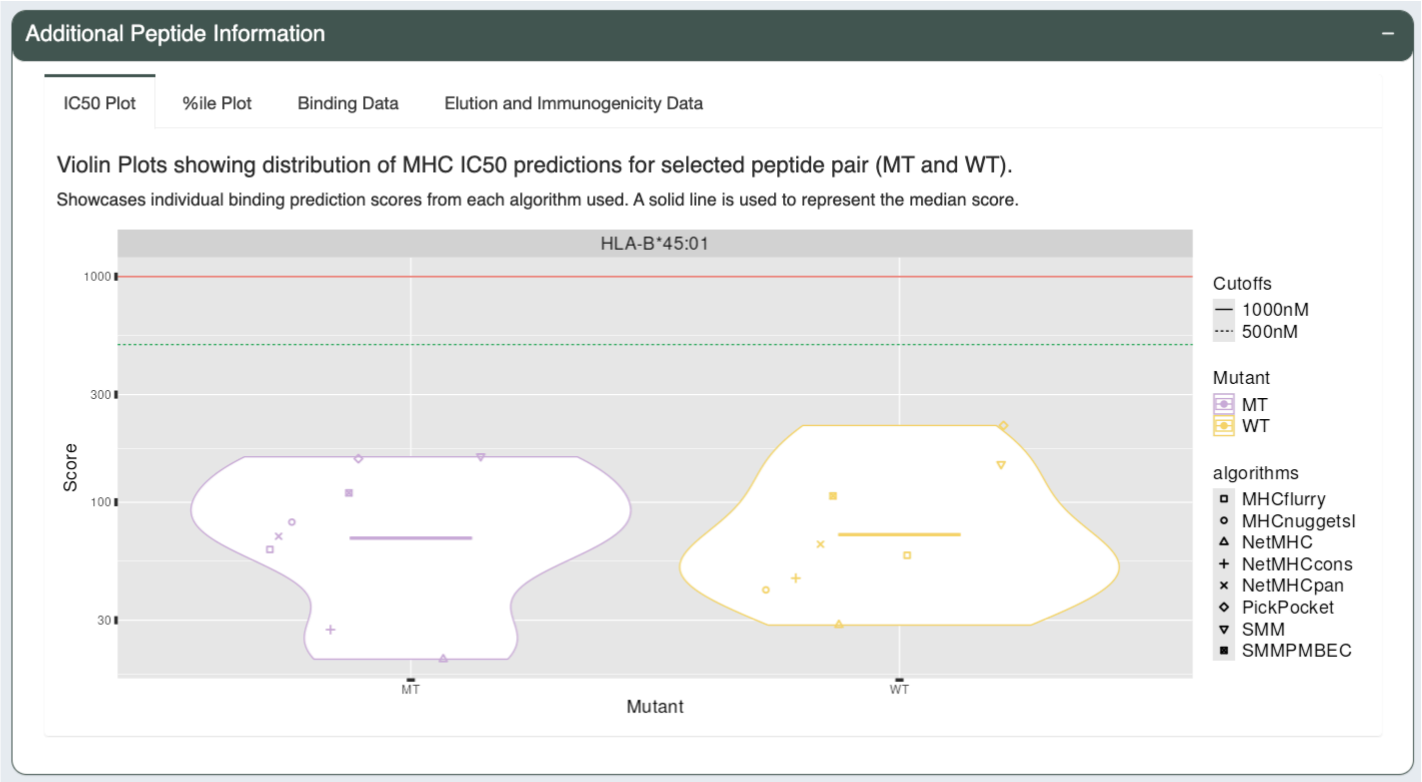

Fig. S7. Visualize and explore - Evaluate an individual peptide: percentile rank (%ile) violin plot
For a selected mutant peptide the %ile plot shows violin plots of the individual algorithm percentile ranks of the MT (mutant) and matched WT (wildtype) peptides for each MHC allele meeting a binding affinity threshold.

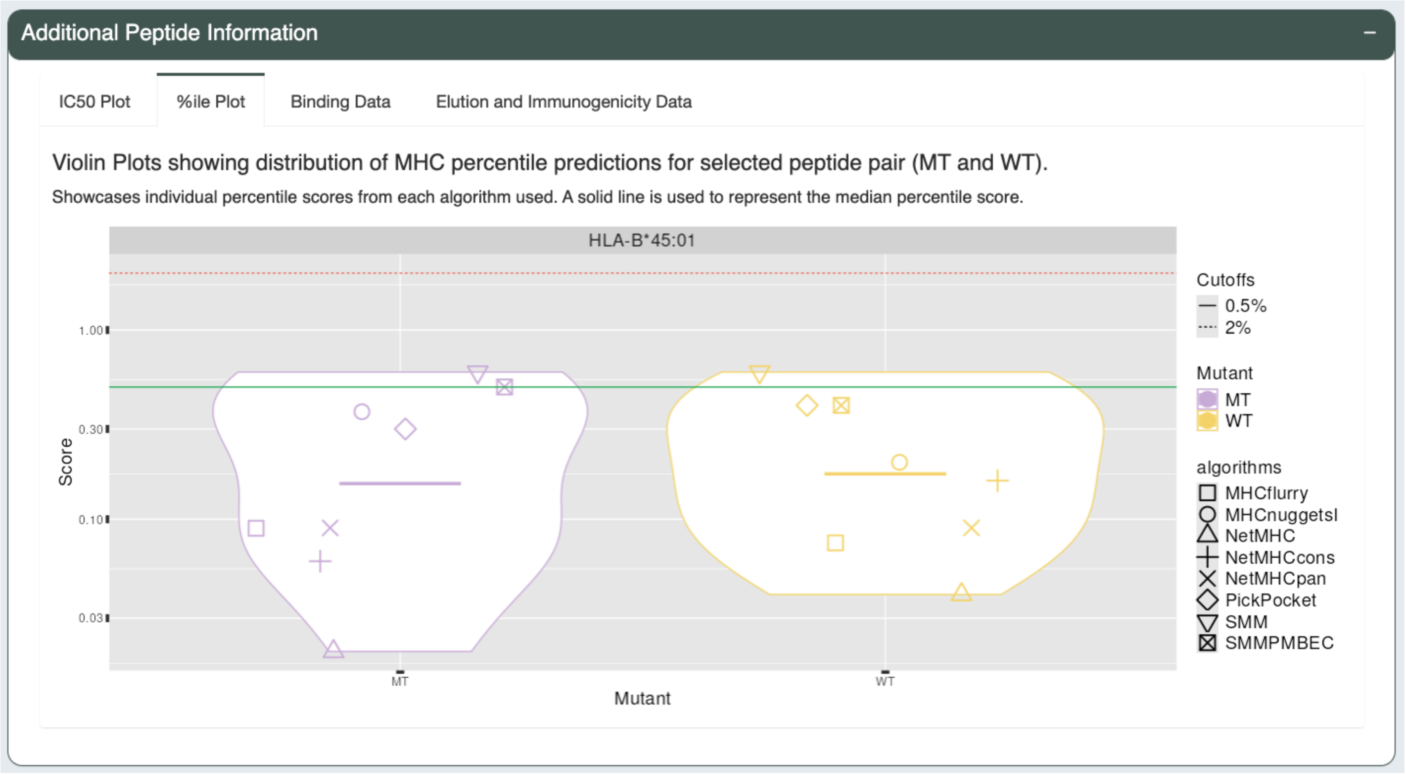

Fig. S8. Visualize and explore - Evaluate an individual peptide: IC50 binding affinity and percentile rank
The Binding Data table shows the numerical IC50 binding affinity and percentile rank values for a selected pair of mutant and wild type peptides across prediction algorithms.

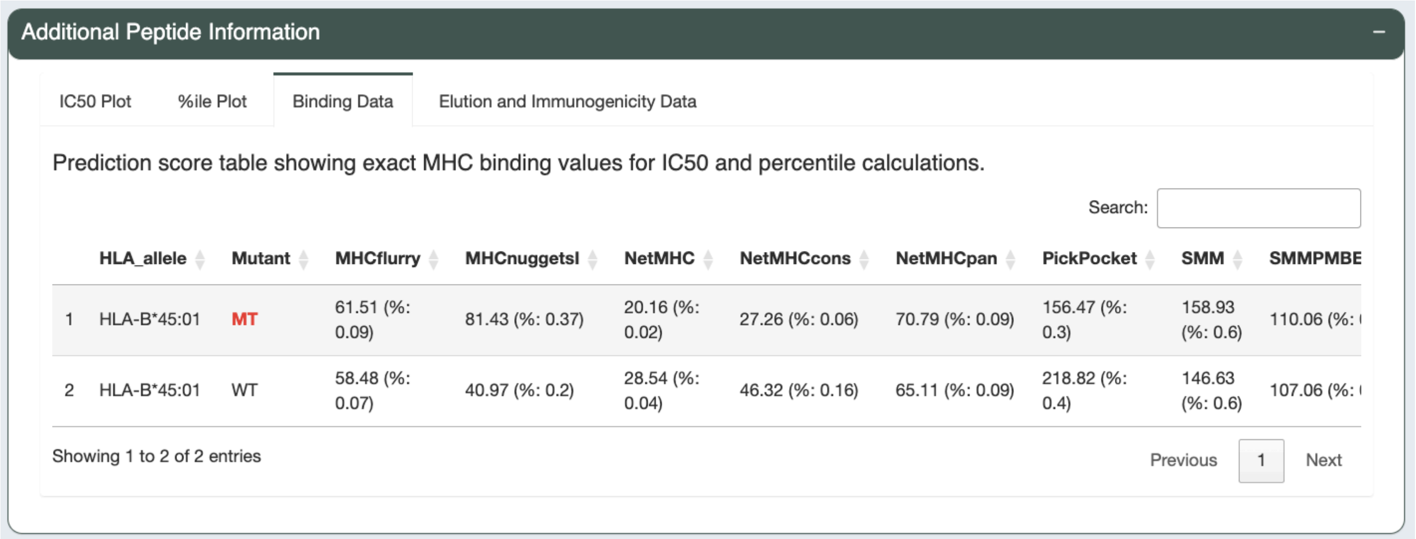

Fig. S9. Visualize and explore - Evaluate an individual peptide: elution and immunogenicity
The Elution and Immunogenicity Data Table lists scores and percentile ranks from predictors trained on peptide elution mass spectrometry data (e.g. BigMHC_EL, MHCFlurryEL, NetMHCPanEL) and immunogenicity data (e.g. BigMHC_IM, DeepImmuno).

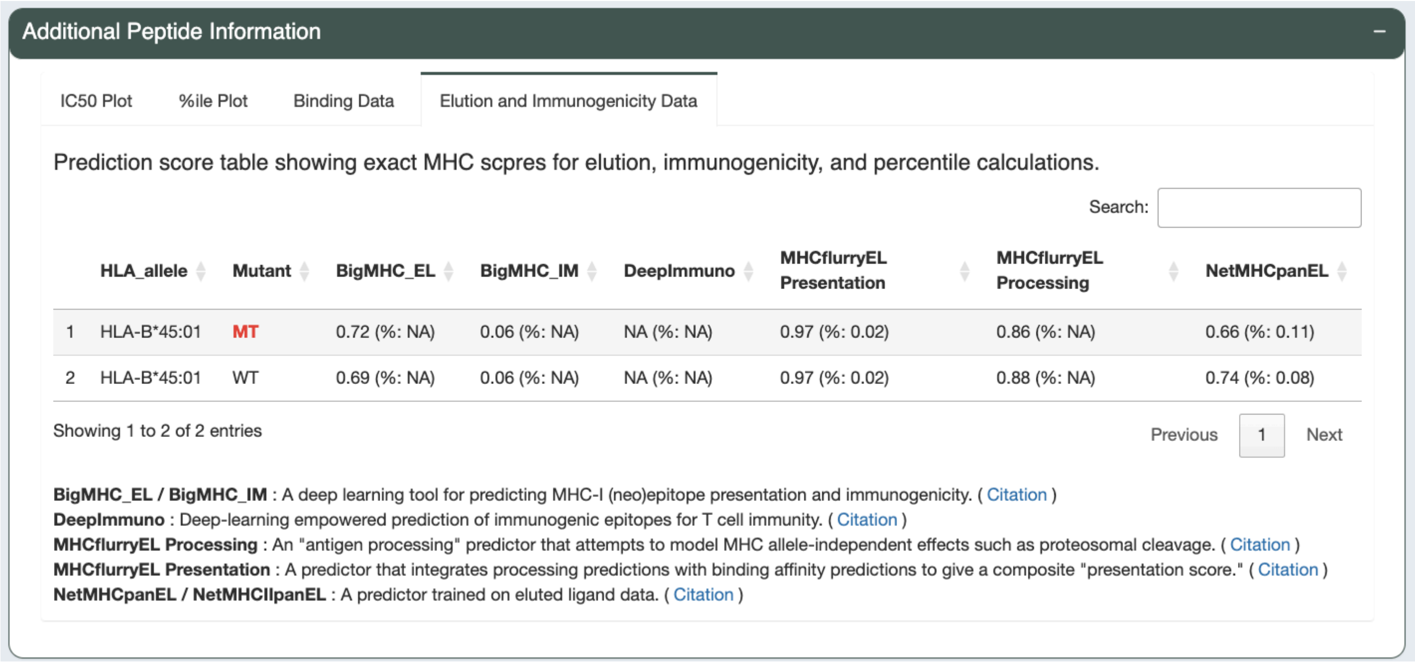
Additional Peptide Information

IC50 Plot | %ile Plot | Binding Data | Elution and Immunogenicity Data

Prediction score table showing exact MHC scpres for elution, immunogenicity, and percentile calculations.

Search:

	HLA_allele	Mutant	BigMHC_EL	BigMHC_IM	DeepImmuno	MHCflurryEL Presentation	MHCflurryEL Processing	NetMHCpanEL
1	HLA-B*45:01	MT	0.72 (%: NA)	0.06 (%: NA)	NA (%: NA)	0.97 (%: 0.02)	0.86 (%: NA)	0.66 (%: 0.11)
2	HLA-B*45:01	WT	0.69 (%: NA)	0.06 (%: NA)	NA (%: NA)	0.97 (%: 0.02)	0.88 (%: NA)	0.74 (%: 0.08)

Showing 1 to 2 of 2 entries

Previous 1 Next

BigMHC_EL / BigMHC_IM : A deep learning tool for predicting MHC-I (neo)epitope presentation and immunogenicity. (Citation)
DeepImmuno : Deep-learning empowered prediction of immunogenic epitopes for T cell immunity. (Citation)
MHCflurryEL Processing : An "antigen processing" predictor that attempts to model MHC allele-independent effects such as proteosomal cleavage. (Citation)
MHCflurryEL Presentation : A predictor that integrates processing predictions with binding affinity predictions to give a composite "presentation score." (Citation)
NetMHCpanEL / NetMHCIIpanEL : A predictor trained on eluted ligand data. (Citation)

Fig. S10. Visualize and explore - Evaluate an individual peptide: anchor residue (heatmap)
For each peptide, we also provide users with an allele-specific anchor prediction heatmap. These predictions are normalized probabilities representing the likelihood of each position of the peptide to participate in anchoring to the MHC molecule. The top 15 MT/WT peptide pairs from the peptide table are shown with anchor probabilities overlaid as a heatmap (darker blue indicating higher probability of the position acting as an anchor). The anchor probabilities shown are both allele and peptide length specific. In the anchor heatmap view, the mutated amino acids are marked in red and MT/WT pairs are separated using a dotted line.

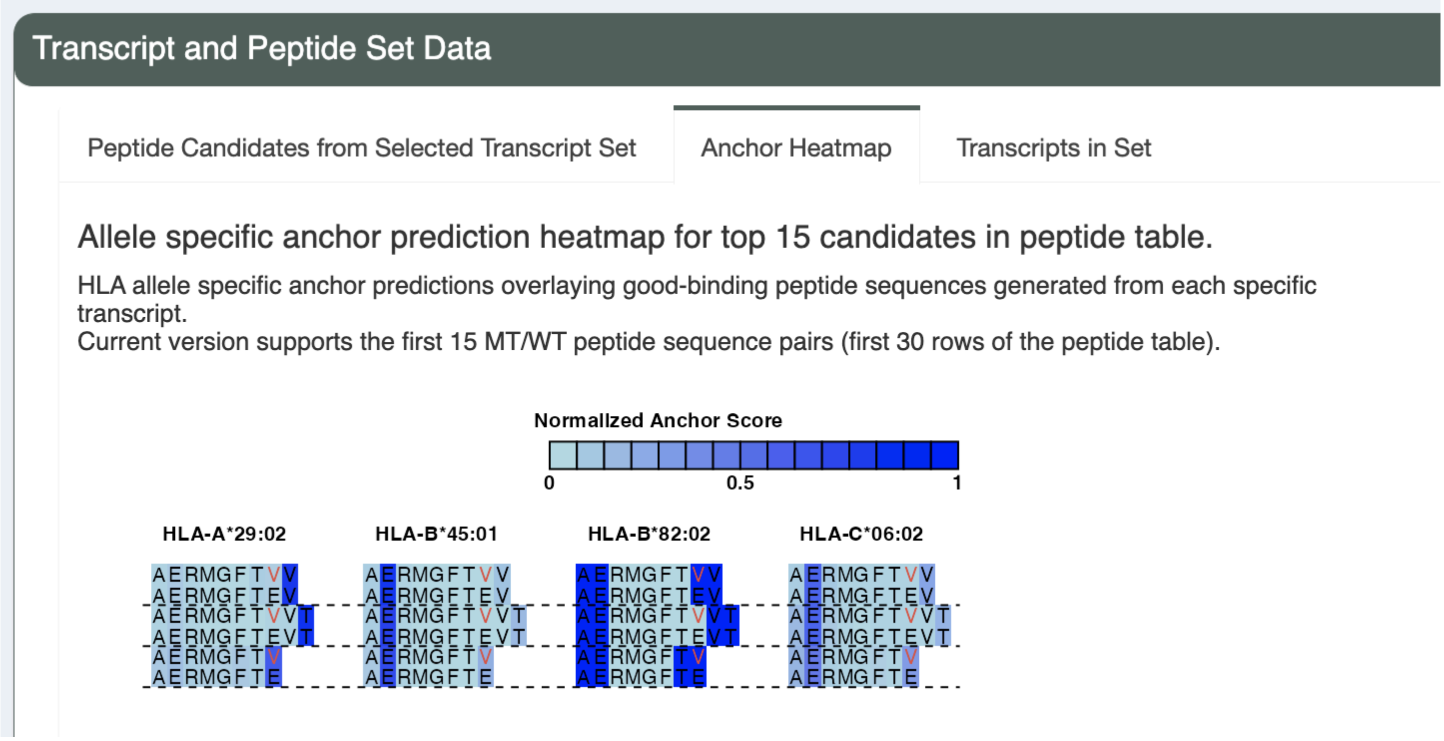

Fig. S11. Visualize and explore - Evaluate an individual peptide: anchor residue (tables)
The list of likely anchor positions and the underlying per-position anchor probabilities can be found in tables at the bottom of the ‘Anchor Heatmap’ tab. Empty entries in the ‘Anchor Weights’ table denote that there is no data available for this allele-length combination.

Anchor Positions –

Search:

	Allele	Length	Anchor Positions
1	HLA-A*29:02	8	1, 2, 6, 8
2	HLA-A*29:02	9	2, 9
3	HLA-A*29:02	10	10
4	HLA-A*29:02	11	11
5	HLA-A*29:02	12	1, 2, 11, 12
6	HLA-B*45:01	8	2, 8
7	HLA-B*45:01	9	2
8	HLA-B*45:01	10	10, 2
9	HLA-B*45:01	11	11, 2
10	HLA-B*45:01	12	1, 2, 11, 12

Showing 1 to 10 of 20 entries

Previous 1 2 Next

Anchor Weights –

Search:

	HLA Allele	Peptide Length	1	2	3	4	5	6	7	8	9	10	11
1	HLA-A*29:02	8mer	0.068141265	0.11413080	0.045241486	0.033563310	0.031645104	0.058932922	0.046453343	0.60189177			
2	HLA-A*29:02	9mer	0.027173719	0.05973064	0.027878104	0.007256377	0.007553450	0.007749724	0.041285151	0.031141772	0.79023106		
3	HLA-A*29:02	10mer	0.018743290	0.06022124	0.024717122	0.007868624	0.009926700	0.008592144	0.008564768	0.011940418	0.015568683	0.83385701	
4	HLA-A*29:02	11mer	0.017371246	0.04931307	0.016605171	0.006899248	0.011749747	0.008182757	0.018084063	0.008777135	0.014420077	0.020995273	0.82760221
5	HLA-B*45:01	8mer	0.063369216	0.69451558	0.018411695	0.005718064	0.009289562	0.007436781	0.009392275	0.19186683			
6	HLA-B*45:01	9mer	0.042517027	0.81338246	0.013212502	0.003560292	0.005820929	0.008049169	0.006774270	0.007645567	0.09903779		
7	HLA-B*45:01	10mer	0.040791470	0.74175530	0.023230564	0.006504404	0.007973135	0.008352411	0.007384598	0.009207378	0.008486203	0.14631454	
8	HLA-B*45:01	11mer	0.041115710	0.74974750	0.011650547	0.005554250	0.004031492	0.007538834	0.008083128	0.009255211	0.009941918	0.016693019	0.13638839
9	HLA-B*82:02	8mer											
10	HLA-B*82:02	9mer											

Showing 1 to 10 of 16 entries

Previous 1 2 Next

Fig. S12. Visualize and explore - Evaluate individual peptide: anchor scenarios
Given the anchor position prediction, users can consult the anchor scenario view (in the 'Anchor Heatmap' tab) to prioritize (Accept) neoantigen candidates. To be specific:
Scenario 1 shows the case where the WT is a poor binder and the MT peptide is a strong binder, containing a mutation at an anchor location. Here, the mutation results in a tighter binding of the MHC and allows for better presentation and potential for recognition by the TCR. As the WT does not bind (or is a poor binder), this neoantigen remains a good candidate since the sequence presented to the TCR is novel. **Scenario 2** and **Scenario 3** both have strong binding WT and MT peptides. In **Scenario 2** , the mutation of the peptide is located at a non-anchor location, creating a difference in the sequence participating in TCR recognition compared to the WT sequence. In this case, although the WT is a strong binder, the neoantigen remains a good candidate that should not be subject to central tolerance. However, as shown in **Scenario 3**, there are neoantigen candidates where the mutation is located at the anchor position and both peptides are strong binders. Although anchor positions can themselves influence TCR recognition, a mutation at a strong anchor location generally implies that both WT and MT peptides may present the same residues for TCR recognition. As the WT peptide is a strong binder, the MT neoantigen, while also a strong binder, will likely be subject to central tolerance and should not be considered for prioritization. **Scenario 4** shows the case where the WT is a poor binder and the MT peptide is a strong binder. In this case, the mutation is located at a non-anchor position, likely resulting in a different set of residues presented to the TCR and thus making the neoantigen a good candidate.

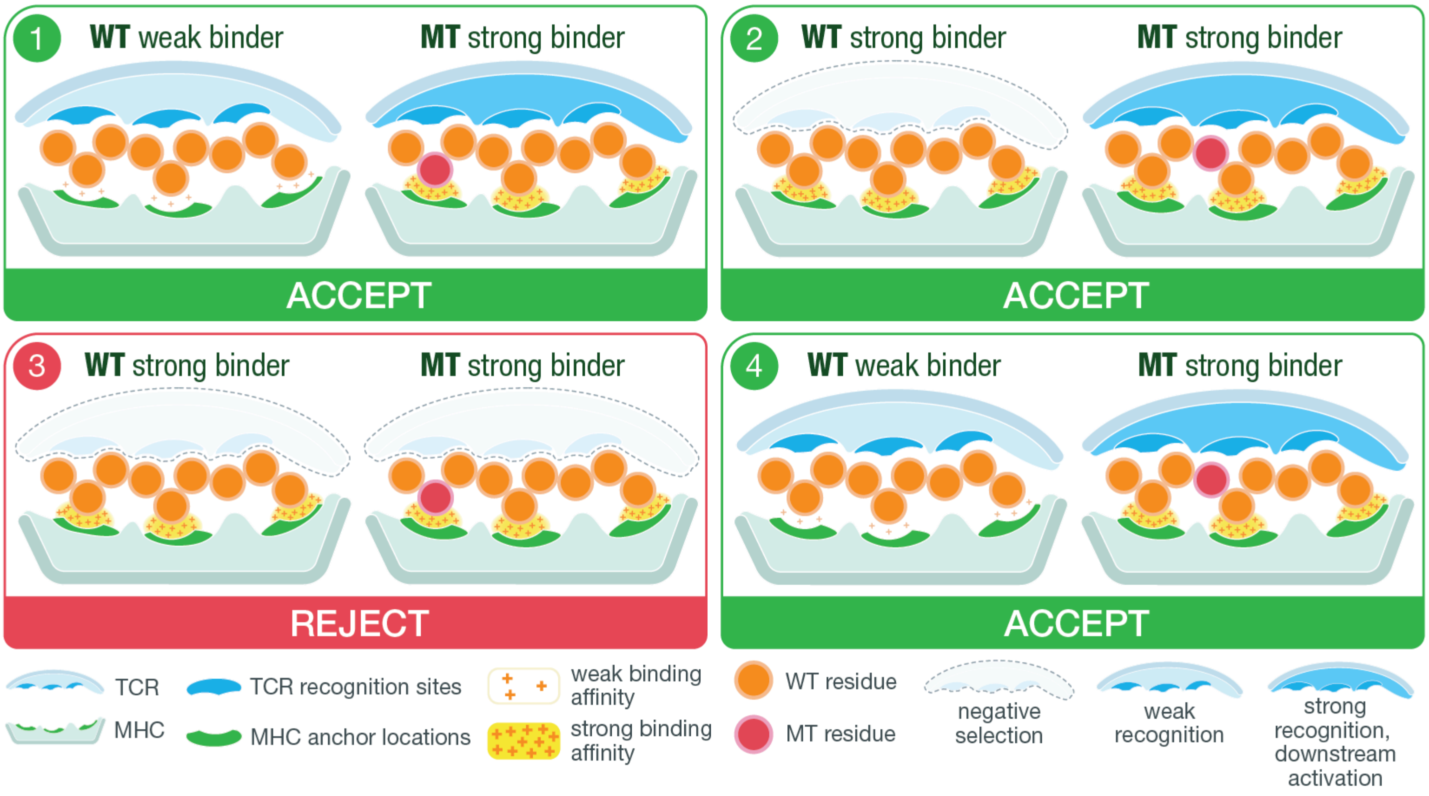

Fig. S13. Visualize and explore - Evaluate problematic features: reference proteome similarity
To ensure that the candidate is a non-self peptide, users can also check if the sequence of the best peptide candidate for a variant matches any sequence found in the reference proteome. This view shows the best peptide with the mutated position(s) highlighted in red as well as the reviewed variant information. The Query Sequence corresponds to a longer sequence around the mutation that is used to search for reference matches. The best peptide is highlighted in yellow in the query sequence. Any 8-mer or longer sub-sequence of the query sequence found in the reference proteome is considered a match and displayed in the table below, along with its gene, transcript, and protein information.

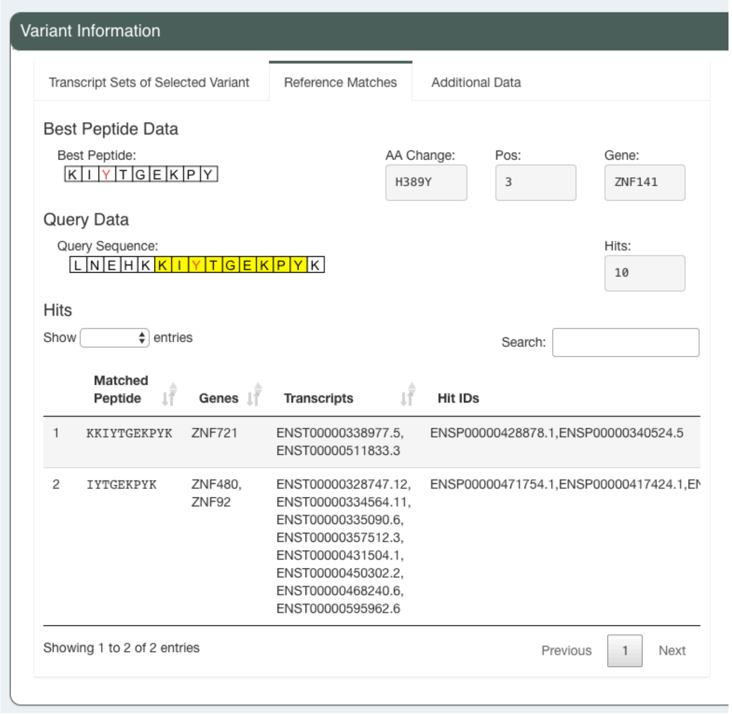

Fig. S14. Visualize and explore - Evaluate problematic features: problematic amino acids
A) If the user specified potential problematic amino acids when running pVACseq, candidates with these problematic amino acids will be flagged by a red box in the “Prob Pos” (Problematic Positions) column in the main aggregate report table. For example, here the user specified Cysteine (C) as a problematic amino acid for manufacturing, and as a result, the neoantigen candidate SRNCEIWQV (with a Cysteine at position 4) is flagged by pVACview. B) This information is also displayed in the Peptide Candidates from Selected Transcript Set table mentioned earlier.

A. Problematic positions highlighted in the aggregate report table

Aggregate Report of Best Candidates by Variant

Number of variants displayed per page:

10

Column visibility

Search:

	Gene	AA Change	Best Peptide	TSL	Allele	Pos	Prob Pos	Num Passing Peptides	IC50 MT	IC50 WT	%ile MT	%ile WT	RNA Expr	RNA VAF	Allele Expr	RNA Depth	DNA VAF	Tier	Ref Match
31	PLAAT4	FS113-120	SRNCEIWQV	1	HLA-C*06:02	6-9	4	1	153.975	5546.497	0.36	5.750	43.703	0.115	5.026	426	0.071	Subclonal	False
32	PARP12	VIFE662-665E	SIFEKHQVY	1	HLA-A*29:02	3-4	None	1	126.8	144.030	0.265	0.295	29.767	0.153	4.554	307	0.108	Subclonal	False
33	DLGAP5	S202L	MPTLLRMTRSA	1	HLA-B*82:02	4	None	1	466.68	434.980	0.73	0.670	53.642	0.224	12.016	825	0.240	Subclonal	False

B. Problematic positions highlighted in the peptide candidates table for a selected variant

Transcript and Peptide Set Data

Peptide Candidates from Selected Transcript Set | Anchor Heatmap | Transcripts in Set

Show 10 entries

Search:

	Peptide Sequence	Type	HLA-A*29:02	HLA-B*45:01	HLA-B*82:02	HLA-C*06:02	Mutation Position	Problematic Positions	Anchor Residue Fail
1	SRNCEIWQV	MT	X	X	X	153.98	6-9	4	None
2	SRNCEHFVT	WT	X	X	X	5546.5	6-9		

Showing 1 to 2 of 2 entries

Previous 1 Next

Fig. S15. Visualize and explore - Capture evaluation status

A) After reviewing each neoantigen candidate, users can leave their evaluation by clicking the appropriate button on the right of each row: thumbs-up for accept, thumbs-down for reject, or flag for candidates requiring further review. For example, a candidate with a reference proteome match might be rejected while a candidate with poor percentile rank might require further review, depending on the user's selection criteria . B) The total number of candidates for each evaluation status are captured in the Peptide Evaluation Overview panel under the 'Variant Information' section.

A. Aggregate Report of Best Candidates by Variant with evaluations selected

Aggregate Report of Best Candidates by Variant

Column visibility ▼

Search:

	Gene	AA Change	Best Peptide	TSL	Allele	Pos	Prob Pos	Num Passing Peptides	IC50 MT	IC50 WT	%ile MT	%ile WT	RNA Expr	RNA VAF	Allele Expr	RNA Depth	DNA VAF	Tier	Ref Match	Acpt	Rej	Rev
11	SIX4	E23Q	QENGMQSA	1	HLA-B*45:01	6	None	2	222.02	324.685	0.23	0.405	15.017	0.987	14.822	76	1.000	Pass	False			
12	ZNF548	D12Y	VVFEYVAIY	1	HLA-A*29:02	5	None	5	41.063	75.490	0.115	0.260	13.433	0.378	5.078	127	0.468	Pass	False			
13	MYBBP1A	E653G	VEVLVGILLA	1	HLA-B*45:01	6	None	1	331.25	403.175	0.73	1.030	47.493	0.319	15.150	420	0.352	Pass	False			
14	UQCC1	W44S	SRTSQSPQM	1	HLA-C*06:02	6	None	1	365.305	752.965	0.594	1.064	47.722	0.327	15.605	581	0.434	Pass	False			
15	SORBS3	R336P	APSLSPHKM	1	HLA-B*82:02	2	None	2	250.848	23477.210	0.37	22.000	23.107	0.374	8.642	195	0.464	Pass	True			
16	ADPRHL1	H220N	QENWFYFEA	1	HLA-B*45:01	3	None	4	11.08	11.610	0.027	0.033	2.743	0.917	2.515	24	0.517	Pass	False			
17	SETD6	R185H	DLANIHSEY	1	HLA-A*29:02	6	None	1	103.73	277.900	0.483	0.940	11.622	0.340	3.951	100	0.335	Pass	False			
18	ATP7A	D870H	HESLITGEA	1	HLA-B*45:01	1	None	1	164.31	360.225	0.4	0.750	5.153	0.951	4.901	41	0.982	Pass	False			
19	ZBTB3	S405F	EPLYLSFEY	1	HLA-A*29:02	7	None	1	382.42	2333.530	1.167	2.800	13.703	0.601	8.236	148	0.670	Pass	False			
20	ZNF25	E21K	KEKWKLLTPA	1	HLA-B*45:01	3	None	1	377.255	137.860	0.64	0.470	13.090	0.471	6.165	187	0.485	Pass	False			

Showing 11 to 20 of 321 entries Show 10 entries

Previous 1 2 3 4 5 … 33 Next

Currently investigating row:

20

B. Peptide Evaluation Overview table

Peptide Evaluation Overview

Evaluation	Count
Accept	8
Pending	311
Reject	1
Review	1

Fig. S16. Visualize and explore - Rescuing poor candidate

In the case that users upload Class I predictions (tsv) as the required input and Class II predictions (tsv) as the additional input, candidates with class I peptides that don't meet a user's selection criteria might be rescued by evaluating the class II peptides, which can be viewed in the Additional Data tab. For example, this TXNDC15 variant (A) has class I peptides with poor IC50 binding affinity, resulting in a Poor tier. However, other selection criteria like the RNA VAF, allele expression, etc. are within range. Moreover, the Additional Data for this variant (B) indicates good class II IC50 binding affinity and percentile rank, therefore, users may consider rescuing this candidate. After leaving a comment (C) on the variant to capture this observation, the candidate is flagged as requiring further review as indicated by the flag button (A).

A. Candidate with poor class I binding affinity and percentile rank

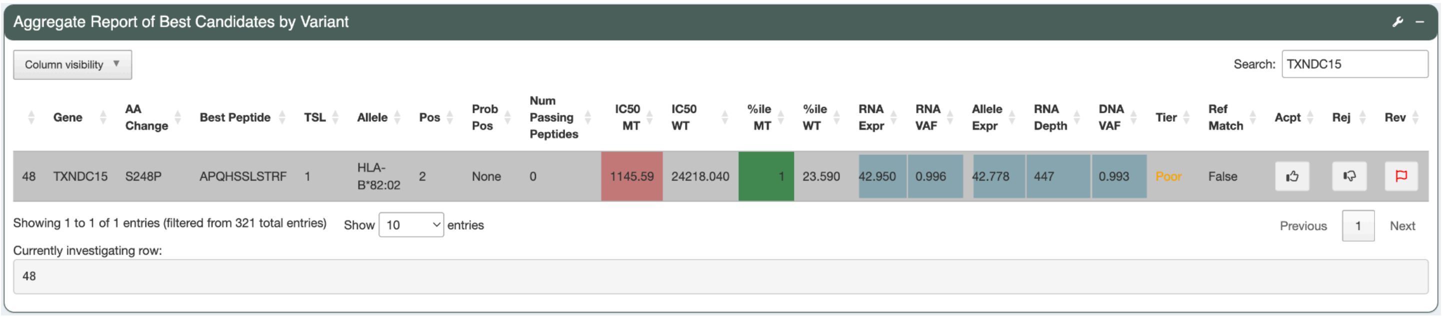

B. Additional class II data for this variant

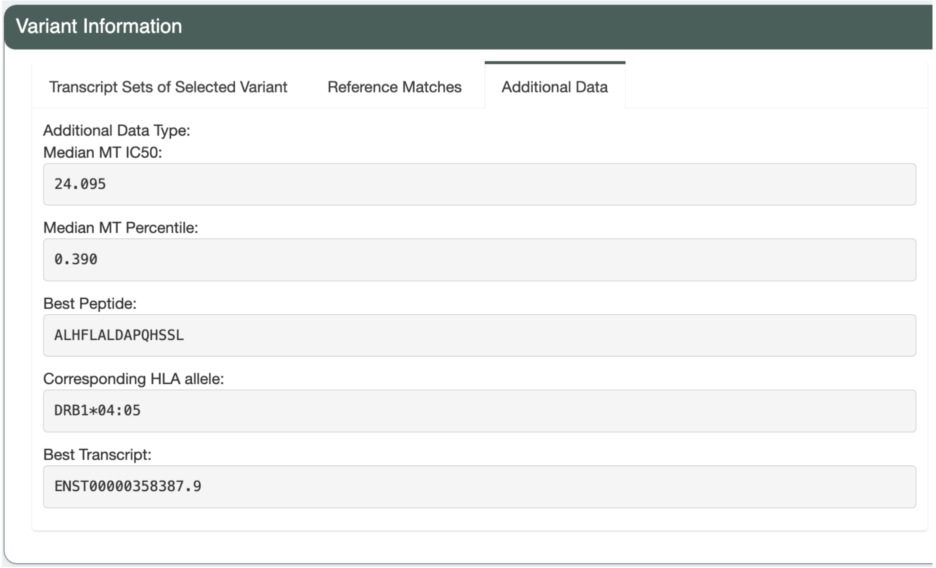

C) Comment interface to capture additional observations for this variant

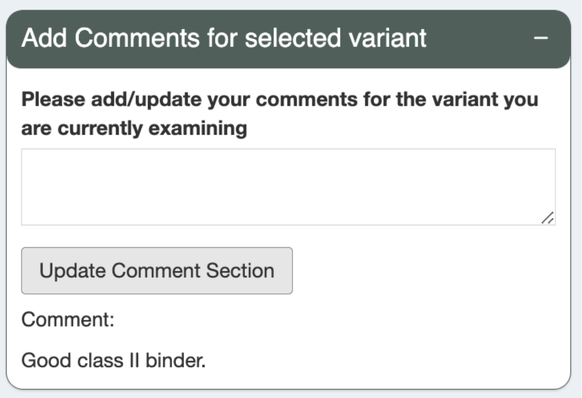

Fig. S17. Export evaluated neoantigens

When users have either finished ranking neoantigen candidates or need to pause and would like to save current evaluations, they can export the current main aggregate report using the export page. We provide two download file types (tsv and excel). The output files include the 'Evaluation' and 'Comment' columns, which capture the evaluations (Accept/Reject/Review/Pending) and comments recorded during the review process.

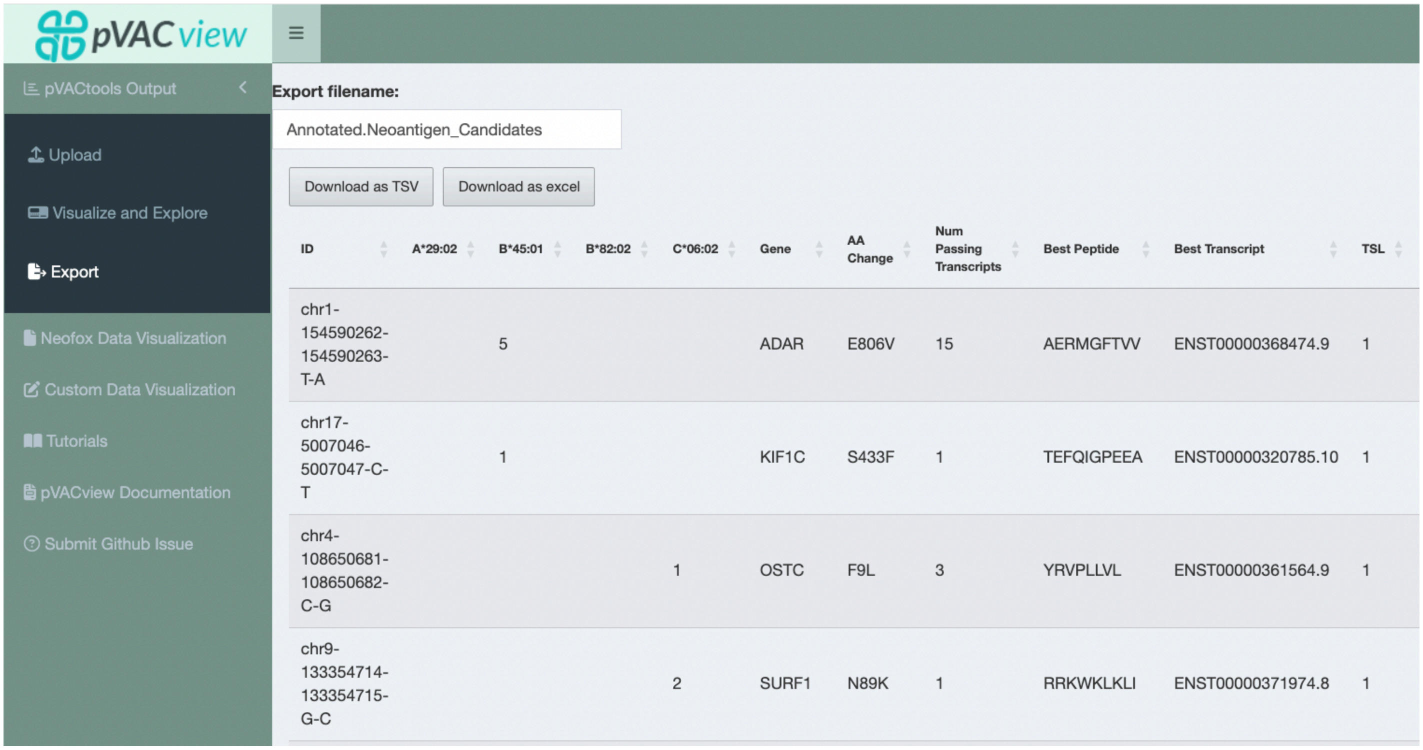

Supplement: Supplementary file 3 — Additional file 3. pVACview main module feature overview. Screenshots and descriptions of all features in pVACview main module. [file 13073_2024_1384_MOESM3_ESM.pdf]
